# Revalidating KCrPDTA for aqueous redox flow batteries

**DOI:** 10.1039/d6eb00156d

**Published:** 2026-09-14

**Authors:** Graham Kimbell, Qirui Liu, Jeonghoon Ahn, Lina Camenisch, Rohat Celik, Talia Echeverria, Baban Deep Virk, Corsin Battaglia, David Reber

**Affiliations:** a Empa, Swiss Federal Laboratories for Materials Science and Technology 8600 Dübendorf Switzerland david.reber@empa.ch; b ETH Zürich, Department of Materials 8093 Zürich Switzerland; c University of Bern, Department of Chemistry, Biochemistry and Pharmaceutical Sciences 3012 Bern Switzerland; d ETH Zürich, Department of Information Technology and Electrical Engineering 8092 Zürich Switzerland; e EPFL, School of Engineering, Institute of Materials 1015 Lausanne Switzerland

## Abstract

Inconsistent reporting practices and single-cell performance claims complicate the comparison of redox flow battery electrolytes across laboratories. Reproducing reported performance metrics for KCrPDTA-based batteries proved challenging, motivating systematic revalidation of KCrPDTA synthesis and characterization, and testing in cells. We identify three compounding variability sources: (i) hydration state inconsistencies in isolated KCrPDTA introducing molar mass errors of up to 4% that propagate into state-of-charge calculations, (ii) impurities eliminated by selective KCrPDTA crystal isolation prior to complete evaporation of the reaction mixture, and (iii) cell assembly artefacts including membrane shorting which produces persistently low coulombic efficiencies unrelated to electrolyte properties. Using a standardized protocol across 22 fuel-cell-type KCrPDTA-K_4_[Fe(CN)_6_] cells, assembled by multiple operators using various material batches, we obtain reproducible average coulombic and energy efficiencies of 99.54 ± 0.08% and 86.3 ± 1.7% over 50 cycles to 80% nominal state-of-charge at 100 mA cm^−2^, values that are statistically grounded in a way single-cell studies are not. Across four cell architectures, coulombic efficiency stays within 99.3 to 99.7% while energy efficiency ranges from roughly 80 to 89%, highlighting the impact of cell hardware on key performance metrics. To support transparent benchmarking, we share all underlying data in a machine-readable format with semantically annotated linked metadata, alongside an open-source analysis pipeline and a metadata reporting template.

## Abstract 2

Single-cell flow battery efficiencies are rarely reproducible. Characterizing many independent KCrPDTA cells under one standardized protocol yields better material understanding, a robust benchmark, and open FAIR data.

## Graphical abstract

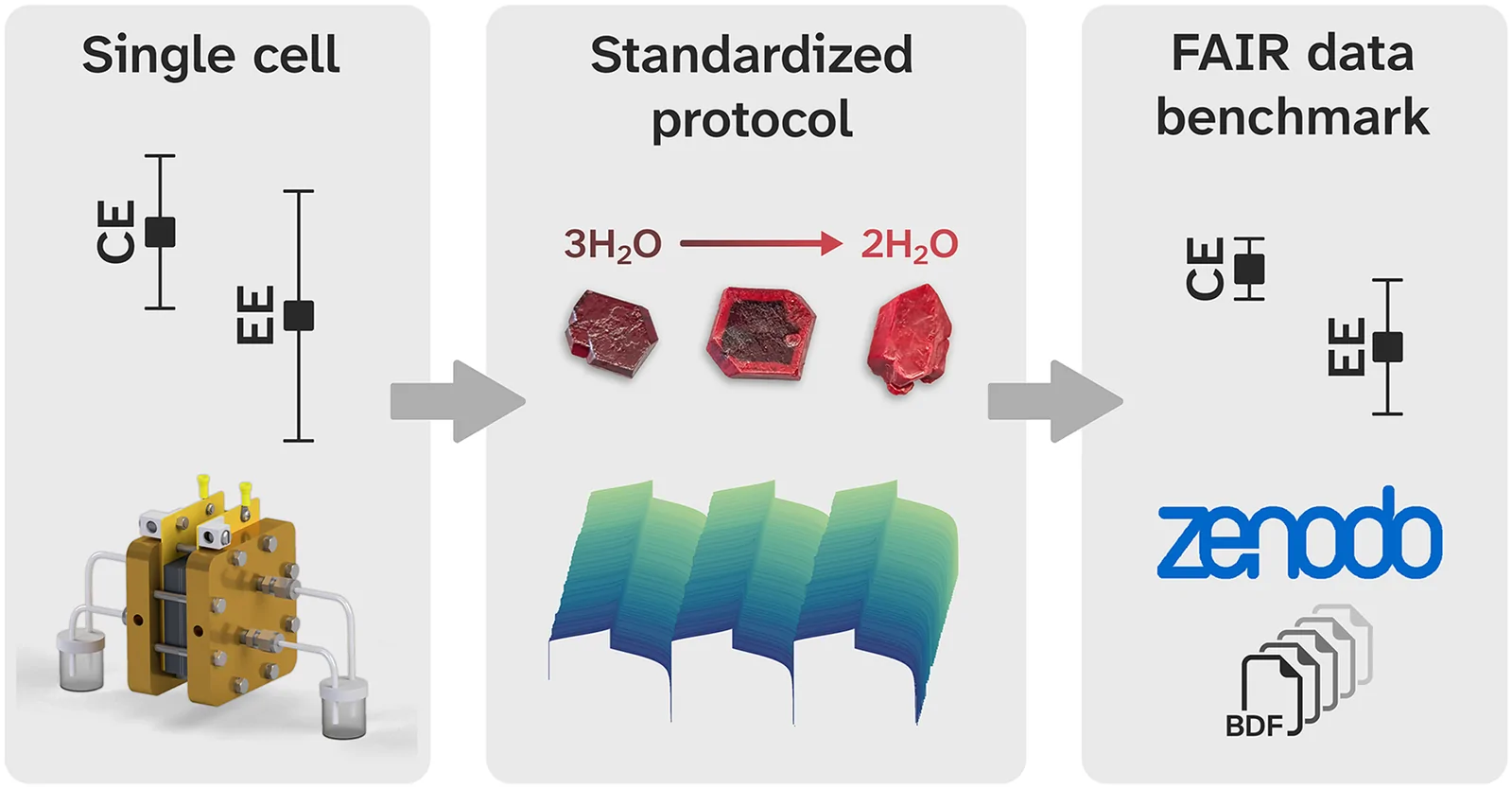

**Broader context**

Redox flow batteries (RFBs) are increasingly deployed for grid-scale storage, but meaningful progress towards lower-cost, high-performance materials is hindered by a reproducibility gap across the RFB literature. This work identifies compounding sources of variability for KCrPDTA, a next-generation negative electrolyte material, and separates true electrolyte performance from hardware artefacts that are easily misattributed. By benchmarking 38 independently assembled cells across multiple operators, material batches, and cell types, we establish a statistically grounded performance baseline that single-cell studies cannot provide and show that KCrPDTA is chemically robust and reusable across cycling campaigns. Beyond this specific chemistry, the practical lessons and the accompanying open, semantically annotated datasets and metadata reporting template apply broadly across RFB electrolyte development, addressing a structural gap where reporting standards lag behind other battery technologies and supporting reproducibility and cross-study comparison.

## 1. Introduction

Aminopolycarboxylate complexes of transition metals^1,2^ capitalizing on the chelate effect^3^ have emerged as a promising class of redox flow battery (RFB) active materials.^4–6^ Chelating transition metal cations with multidentate ligands can drastically modify the pH stability, redox potential, reaction kinetics, and solubility of redox-active metal centers relevant to electrochemical energy storage, and aminopolycarboxylates directly provide strong binding motifs capable of stabilizing highly reducing species across a broad pH window.^7^

In the case of chromium, among the first materials considered for RFBs,^8^ coordination geometry plays a decisive role.^9,10^ Chelation with ethylenediaminetetraacetate (EDTA) yields a pentacoordinate complex in which one carboxylate arm remains unbound in pH-neutral solution, permitting inner-sphere water coordination to the chromium center, catalyzing hydrogen evolution upon reduction of the CrEDTA^−^ complex.^11^ Extending the ligand backbone by a methylene unit to form 1,3-diaminopropane-*N*,*N*,*N*′,*N*′-tetraacetate (PDTA^4−^) relieves strain in the equatorial plane of the CrPDTA^−^ complex and enables a hexacoordinate structure that persists in solution and is stable in both the oxidized and reduced Cr(iii)/Cr(ii) states.^11,12^ This structural change further shifts the Cr(iii)/Cr(ii) redox potential to −1.3 V *vs.* Ag/AgCl, compared to −0.6 V *vs.* Ag/AgCl (sat. KCl) for free chromium,^13^ enabling high cell voltage and power densities when coupled with, *e.g.*, ferrocyanide/ferricyanide-based positive electrolytes. Simultaneously, chelation improves redox kinetics, enhances solubility across a wide pH range, minimizes crossover, and suppresses hydrogen evolution by excluding water from the inner coordination sphere of the metal center.^14,15^ Pioneered by the Marshak group, the CrPDTA^−^ chelate has therefore emerged as one of the most promising negative electrolyte materials for high-power, near-neutral-pH aqueous RFBs.^14–22^

The work presented here is motivated by repeated difficulties in reproducing reported CrPDTA^−^ performance metrics. Despite prior experience with the material and direct familiarity with synthesis procedures, cell components, and testing protocols, nominally identical methods reproduced in a new laboratory resulted in lower-than-expected performance. Although the synthesis of KCrPDTA itself is conceptually straightforward, subtleties in purification, cell assembly, and testing conditions can compound to affect the coupled processes in complex systems such as RFBs.^23^ Here, we distill practical lessons learned from revalidating KCrPDTA as a benchmark negative electrolyte for next-generation RFBs and provide benchmark data with semantically annotated linked metadata to promote transparency and facilitate interlaboratory comparison.

## 2. Methods

### 2.1 Materials

Potassium ferrocyanide trihydrate (>99%, K_4_[Fe(CN)_6_]·3(H_2_O)), potassium ferricyanide (>99%, K_3_[Fe(CN)_6_]), chromium(iii) sulfate hexahydrate (reagent grade, Cr_2_(SO_4_)_3_·6(H_2_O)), and potassium hydroxide (>85%, pellets, KOH) were purchased from Fisher Scientific and used as received. 1,3-Diaminopropane-*N*,*N*,*N*′,*N*′-tetraacetic acid (>99%, H_4_PDTA) was purchased from Sigma Aldrich and used as received. Chromium(iii) potassium sulfate dodecahydrate (>98%, KCr(SO_4_)_2_·12(H_2_O)) was purchased from Fisher Scientific and recrystallized before use to remove yellow and magnetic grey impurities. Aqueous solutions were prepared using ultrapure water (Milli-Q EQ 7000, 18.2 MΩ cm at 25 °C).

### 2.2 KCrPDTA synthesis

0.1 mol KCr(SO_4_)_2_·12(H_2_O) (49.94 g) and 0.1 mol H_4_PDTA (30.60 g) were added to a 1 L round bottom flask followed by 300 mL ultrapure water. Note that precursor purity was not considered in the calculation, resulting in an effective ligand excess of approximately 1%. The mixture was brought to reflux (oil bath set to 125 °C), turning clear and increasingly red above *ca.* 80 °C. The solution was refluxed for 48 hours and stirred at 700 rpm. Four equivalents of KOH, 100 mL 4 M KOH (0.34 mol considering KOH purity), were then added at a rate of 2.10 mL h^−1^ (∼48 h) using two syringe pumps equipped with 60 mL syringes. The mixture was cooled to room temperature, and the pH was recorded as 1.4. Additional KOH was added until the pH reached a value of 5.2, requiring 45 mL of 2 M KOH or 0.9 equivalents (0.08 mol considering KOH purity). The pH was monitored for an additional hour to confirm that it remained stable. The final solution volume was approximately 500 mL. 750 mL acetone were then added to the solution to precipitate solid K_2_SO_4_, which was removed by filtration and washed with 50 mL of a 1 : 4 water : acetone mixture, resulting in a white filter cake. After drying at 80 °C for 24 hours, 34.33 g of K_2_SO_4_ were recovered, corresponding to a by-product yield of 98.5%. The clear red filtrate was left to evaporate at room temperature. When only a few mL of solution were left, the liquid was poured off, and dark red KCrPDTA·3H_2_O crystals were collected, rinsed with ultrapure water, and then left to dry in a fume hood. Thermogravimetric analysis (TGA) and differential scanning calorimetry (DSC) were carried out on a Netzsch STA 449 Jupiter coupled to a QMS 403 C quadrupole mass spectrometer under N_2_ (20 mL min^−1^) between −50 °C and 550 °C at a heating rate of 10 K min^−1^.

### 2.3 Battery electrolytes

10 mL of 1 M KCrPDTA (10 mmol) and 0.1 M PDTA buffer (pH 8.5) in Milli-Q water was used as the negative electrolyte resulting in a solution pH of 8.7–8.8. For the positive electrolyte, 30 mL of 0.5 M K_4_[Fe(CN)_6_] (15 mmol) and 0.1 M K_3_[Fe(CN)_6_] (3 mmol) in water (pH 7.7) were used. The KCrPDTA negative electrolyte is therefore capacity-limiting, so that the reported capacities and coulombic efficiencies reflect the negative electrolyte under study rather than being influenced by [Fe(CN)_6_]^4−^ availability. State-of-charge and the nominal 80% cap refer to the negative electrolyte capacity.

### 2.4 Cell assembly

Two stacked Sigracet 39 AA (SGL Carbon) carbon paper electrodes with an area of 5 cm^2^ were used on each side of a Fumatech E-630(K) cation exchange membrane with a thickness of ∼30 µm. The carbon paper was used as received (no thermal treatment) and cut to shape with a scalpel and a ruler. Flow cells in zero-gap configuration with serpentine flow fields and gold-coated current collectors (Fuel Cell Technologies Inc.) were used for flow battery testing with 0.45 mm thick Viton gaskets (Maagtechnic AG) resulting in 20% electrode compression. The gaskets were notched in one corner for prying open the cell more easily during disassembly, as the gaskets sometimes stick together. The cells were sealed by sequentially tightening the bolts first to 2, 4, then 6 Nm with a torque wrench. For select experiments, cells from Pinflow energy storage s.r.o. or 3D-printed designs were employed. The fuel-cell-type and Pinflow cells use 5 cm^2^ carbon paper electrodes, whereas the 3D-printed cells use 16 cm^2^ carbon felts. Full assembly parameters are provided in the corresponding metadata files (see Data analysis and reporting). LS16 Norprene tubing (3.1 mm inner diameter) and peristaltic pumps (Longer WT600-2J or Watson Marlow 323) were used to circulate the electrolytes.

### 2.5 UV–Vis analysis

The UV–Vis spectroscopy measurements were performed on an ALS SEC2020 UV–Vis Spectrometer which was warmed up for 45 minutes and referenced with water prior to every measurement series. The molar absorptivity of KCrPDTA was determined from 2 mg mL^−1^ solutions of KCrPDTA dihydrate. Beer–Lambert linearity was confirmed with a separate calibration series (0.5, 1, 2, 3, and 5 mg mL^−1^) measured in the same 1 cm quartz cuvette (Starna).

### 2.6 Electrochemical tests

Flow battery cycling experiments were performed using a Biologic BCS-815 battery cycler or SP-150e potentiostat. Prior to each experiment the flow rate of 60 mL min^−1^ was confirmed with the cell in series by circulating water before draining and filling with active electrolytes. For initial experiments the protocol varied, as seen in the raw data. The standardized protocol that was developed and then employed for all tests is as follows: the electrolytes are circulated for 1–2 minutes. The pumps are then stopped and cyclic voltammograms in the capacitive cell voltage range between 0.4 and 0.6 V at increasing scan rates of 10, 25, 50, 75, 100, 125, 150, 200, 250 mV s^−1^, three scans per scan rate, are recorded. The average current between the charge and discharge current at 0.5 V is extracted and plotted against the scan rate to estimate the cell capacitance as a proxy for electrochemically active electrode surface area. The capacitance is obtained from the slope of the linear fit. The pumps were then turned back on and the KCrPDTA solution was degassed by sparging humidified Ar during the entire experiment, which also provided electrolyte agitation. The electrolytes were not separately stirred. The open circuit voltage (OCV) was monitored for one hour, followed by an electrochemical impedance spectroscopy (EIS) measurement in the frequency range from 10 kHz to 50 mHz with a sinus amplitude of 10 mV and 6 points per decade to estimate the ohmic resistance of the cell. Linear sweep voltammetry (LSV) was then performed at 200 mV s^−1^ from OCV to 1.8 V to obtain the initial area specific resistance (ASR) as the inverse of the slope of a linear fit to the LSV curve between 1.6 and 1.8 V. Next, a pre-charge to a nominal 50% state-of-charge, assuming 100% faradaic efficiency, was performed by applying 500 mA for 16 minutes and 5 seconds, followed by an EIS measurement. The cell was then discharged at −500 mA to 0.8 V. The constant-current cycling protocol to probe coulombic efficiency then consisted of 50 cycles to 80% nominal SOC at ±500 mA with potential limits of 0.8 and 2.0 V. Cycling was capped at 80% nominal SOC to keep the cell away from the high-voltage tail of the charge curve, where the negative electrolyte approaches full reduction and side reactions such as water reduction become more favorable. This cap is also consistent with common practice in the KCrPDTA literature, facilitating comparison. The cell was then fully discharged with a 5 minute voltage hold at 0.8 V, followed by the same sequence of charging to 50% nominal SOC, EIS, full discharge, EIS, LSV, full discharge, to enable comparison of pre- and post-cycling cell resistances. All cells were cycled at the same absolute current of ±500 mA, corresponding to 100 mA cm^−2^ for the fuel-cell-type and Pinflow cells and 31.25 mA cm^−2^ for the 3D-printed cells. The lower current density of the 3D-printed cells was chosen to maintain comparable cycle duration across cell architectures and contributes to their higher voltage efficiency.

### 2.7 Data analysis & reporting

With the well-defined testing protocol, we can perform systematic data analysis. We have developed a Python-based tool called ‘PyFlowBatt’ to perform all analysis and present results with one command. The tool takes a folder of raw data as an input. First, we parse data into the ‘Battery Data Format’ (BDF), an open data standard proposed by the Battery Data Alliance, a Linux Foundation Energy project, storing timeseries data either in .csv and/or .parquet format.^24^ Parquet is a widely used, fast and space-efficient file format for columnar data that can be read by many programming languages and tools without proprietary software. BDF defines common column headers so data from different instruments can be represented in a consistent way.

We then iterate through the data files, perform the appropriate analysis, *e.g.*, fitting EIS or calculating coulombic, voltage, and energy efficiencies from cycling data, and output plots, summary data, and processed data. All reported uncertainties denote one standard deviation unless stated otherwise. The tool can also be used on several folders (so several cells) at once and will generate a summary report across all cells. ‘PyFlowBatt’ is open-source, and built on top of other free, open-source projects from the battery community.^24–26^

Timeseries data are accompanied by semantically annotated linked metadata files in .json format describing the cell assembly (electrodes, electrolytes, components) compliant with the BattINFO ontology,^27,28^ a battery domain ontology embedded in the Elementary Multiperspective Materials Ontology (EMMO).^29,30^ To facilitate adoption of findable, accessible, interoperable, reusable (FAIR) data principles in the flow battery community, we created an Excel template for flow batteries that can be converted into a machine-readable linked-data .json metadata file using the open-source BattINFO converter web application^31^ without prior knowledge in ontologies and programming skills. The template can be adapted and used standalone as a structured checklist of what metadata should be reported in RFB studies alongside experimental procedures. We note that this template is offered as one contribution among parallel efforts currently underway in the redox flow battery community to establish consensus reporting standards. We anticipate that continued community engagement, including forthcoming methodology work from other groups, will help converge these efforts toward a widely adopted standard.

## 3. Results and discussion

### 3.1 Material characterization

The first reproducibility challenge is the inconsistency in the reported composition of KCrPDTA used in flow battery electrolytes. Freshly crystallized KCrPDTA forms dark red, hexagonal crystals. Upon storage at room temperature, these crystals become lighter, less brittle, and brick-red in color, while drying at elevated temperatures further induces a purple hue.

**Fig. 1:**
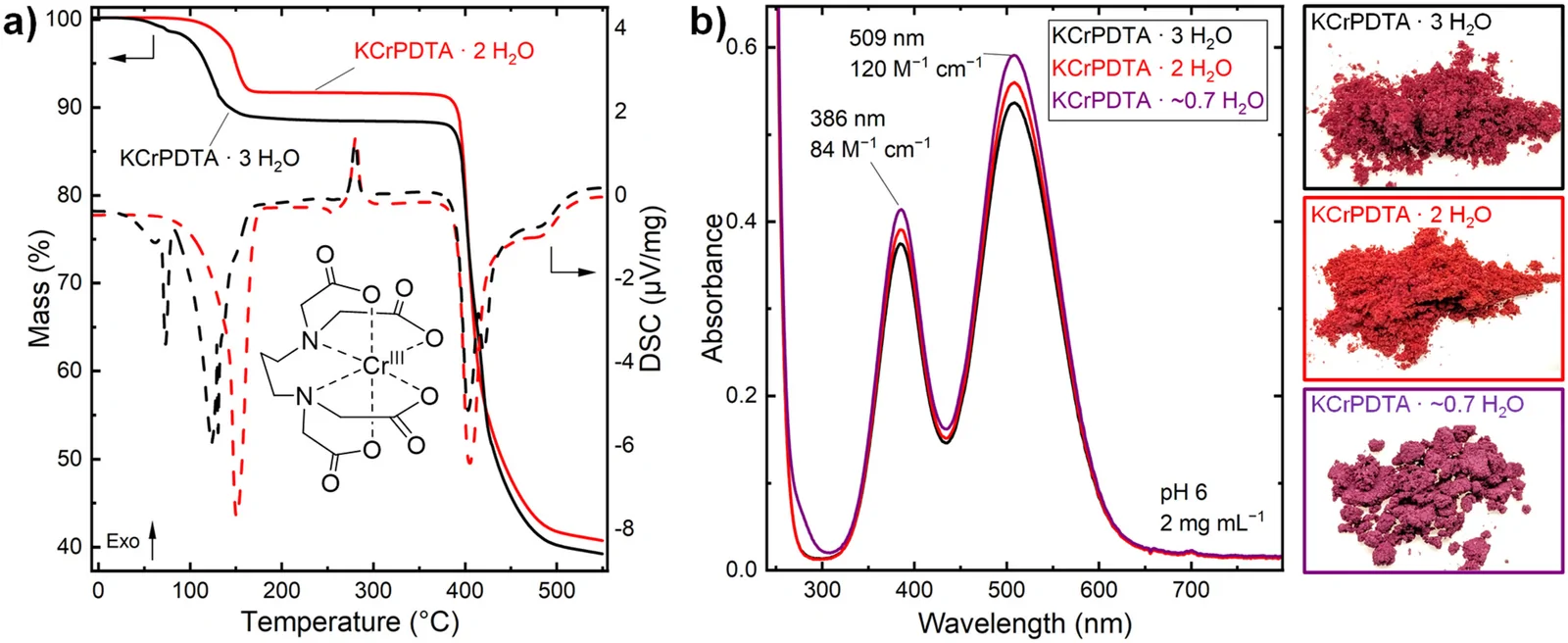
(a) TGA and DSC of KCrPDTA di- and trihydrate, and molecular structure of the CrPDTA^−^ anion. (b) UV–Vis absorption spectra of 2 mg mL^−1^ aqueous solutions of KCrPDTA with varying degrees of hydration, and photographs of the corresponding solid samples.

TGA revealed that these color changes correspond to differences in crystal water content (Fig. 1a). The molar mass of KCrPDTA is 393.3 g mol^−1^. Dark red crystals that were hand-picked from an evaporating KCrPDTA liquor and gently dried with a tissue lost approximately 12 wt% in an endothermic process prior to decomposition above 370 °C, confirming that the initial mass loss corresponds to lattice water rather than ligand degradation (Fig. S1) and indicating an initial molar mass of 447.4 g mol^−1^, consistent with KCrPDTA·3H_2_O and in agreement with the reported crystal structure.^11,12^

Similarly, a brick-red sample dried at room temperature for one week lost 8.5 wt% upon heating, corresponding to an initial molar mass of 429.4 g mol^−1^ consistent with KCrPDTA·2H_2_O. This difference in hydration leads to molar-mass variations of approximately 4%, which directly translate into concentration errors if neglected when electrolyte solutions are prepared gravimetrically. Solutions containing 2 mg mL^−1^ of the trihydrate exhibited *ca.* 4% lower absorbance than those prepared from the dihydrate (Fig. 1b), matching the expected concentration difference arising from hydration. UV–Vis analysis further suggested that material dried at 150 °C for 24 hours still retains approximately 0.7 equivalents of lattice water. Notably, the purple color of this partially dehydrated sample reverted to brick-red upon equilibration under ambient conditions, indicating at least partly reversible hydration of solid KCrPDTA·2-*x*H_2_O.

Samples with such well-defined hydration states allow refinement of the molar absorptivity values for the spin-allowed ^4^A_2g_ → ^4^T_2g_ and ^4^A_2g_ → ^4^T_1g_ transitions of CrPDTA^−^_(aq)_, previously reported as 116–119 M^−1^ cm^−1^ at 506–509 nm and 73–83 M^−1^ cm^−1^ at 382–385 nm.^12,32–34^ Dissolution of accurately weighed KCrPDTA di-/trihydrate in ultrapure water yielded molar absorbance of 120 M^−1^ cm^−1^ at 509 nm and 84 M^−1^ cm^−1^ at 386 nm at pH 6, small yet practically relevant differences when combined with uncertainty in hydration state. Beer–Lambert linearity over the relevant concentration range was confirmed separately (Fig. S2). Because concentrations are typically derived from gravimetric electrolyte preparation in RFB studies, such variability in molar mass directly translates into miscalculated concentrations, theoretical capacities, and state-of-charge windows on the order of 5% and hence potentially misrepresented coulombic efficiency (CE). Note that the absorbance of CrPDTA^−^ varies only weakly between pH 1–10,^11^ yet pH values should nevertheless be reported alongside UV–Vis spectra to enable comparison across studies.

### 3.2 Reported synthesis and battery performance

While hydration variability introduces uncertainty in the effective composition of KCrPDTA electrolytes, further discrepancies arise from differences in material synthesis. Reported procedures for the preparation of the CrPDTA^−^ complex span a range of chromium precursors, reagent addition sequences and rates, reaction durations and temperatures, and workup strategies, often described qualitatively. Such variations are non-trivial in the context of kinetically inert Cr(iii) coordination chemistry, where subtle differences in pH evolution or intermediate speciation can promote hydrolysis, olation, and the formation of poorly defined side phases.^35–37^ Consequently, materials obtained from seemingly similar syntheses may differ in purity, which may impact battery performance.

First reports mix H_4_PDTA, sometimes labelled H_4_TRDTA, with two equivalents of NaOH, followed by heating to 70 °C and addition of one equivalent of Cr(iii) nitrate or chloride. After “heating for a period” two more equivalents of base are added, followed by heating for another 12 h, and subsequent cooling to 0 °C to precipitate red NaCrPDTA crystals.^33,38^ Note that the sodium salt is poorly soluble in water and that this method of separation is not practical for more soluble analogs such as KCrPDTA or LiCrPDTA.^39^ Already in 1970 Ogino *et al.* noted that a heated equimolar solution of Cr(iii) and H_4_PDTA first forms a viscous liquid of a coordination polymer, that only very slowly converts into monomeric CrPDTA^−^ complexes.^34^ Furthermore, the targeted precipitation of the product to remove chloride or nitrate ions is difficult and often ignored in metal–organic RFB literature, even though the presence of such ions can affect solubility, viscosity, speciation, or redox potentials. More recent battery-focused reports have adapted the procedure for KCrPDTA to pound-scale,^18^ applying higher heat for longer periods of time and using KCr(SO_4_)_2_·12H_2_O as the Cr(iii) source. K_2_SO_4_ is formed as a byproduct that can easily be separated by precipitation upon addition of an antisolvent such as acetone to the reaction mixture.

With variations, the following procedure emerges in the RFB literature:^7,12,14,18,21,39,40^ KCr(SO_4_)_2_·12H_2_O and H_4_PDTA are mixed in water at approximately equimolar metal-to-ligand ratio, sometimes with a small ligand excess, and heated to reflux for an extended period, typically 12–24 h to initiate Cr(iii)–ligand coordination. Base, most commonly KOH, is introduced gradually, either as solid portions or as concentrated aqueous solution added in undefined increments. Over 48–72 h total reaction time, additional KOH is added in one or more stages, and the mixture is ultimately adjusted to a mildly acidic endpoint at pH 5–6, sometimes with further heating holds between additions. After cooling to room temperature, a roughly equivalent volume of acetone is added to the red solution to precipitate K_2_SO_4_, which is removed by filtration, leaving a clear red filtrate containing KCrPDTA. Note that the K_2_SO_4_ yield for this procedure has not been reported. The filtrate is concentrated and allowed to evaporate under ambient conditions, resulting in dark red crystals. These are sometimes rinsed with water and dried in air. Reported hydration states and molar masses of the final product vary, consistent with the dehydration behavior discussed above.

**Fig. 2:**
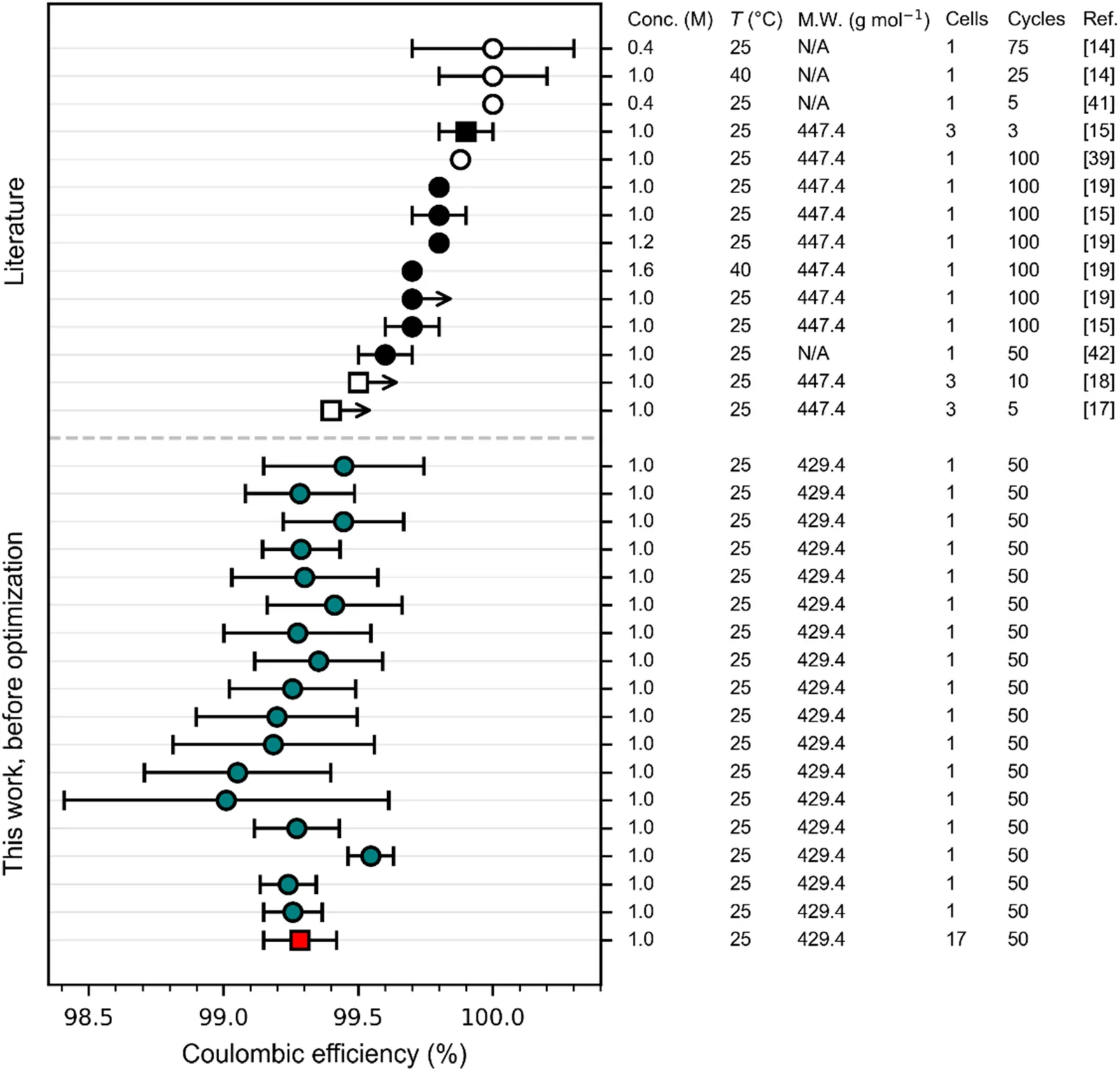
Literature survey of reported coulombic efficiencies for KCrPDTA-K_4_[Fe(CN)_6_] RFBs cycled between 0–80% nominal state-of-charge at 100 mA cm^−2^ and distribution of efficiencies across 17 independently assembled cells (50 cycles each) using optimized membranes and electrolytes in the lead up to this work. The mean per-cycle coulombic efficiency across these cells is shown in red. The cells were assembled following published procedures, prior to the workup, hydration, and assembly refinements developed in this work, and represent our initial revalidation attempts rather than optimized performance. For literature values, arrows indicate values reported as lower bounds (*e.g.*, “>99.4%”). Circles represent single-cell experiments, while squares denote averages over multiple cells. Open symbols correspond to early reports using unoptimized components (*e.g.*, Nafion™ membranes and borate buffers),^14,17,18,39,41^ filled symbols denote cells employing optimized parts (*e.g.*, Fumatech E620-K membranes and ligand-buffered electrolytes).^15,19,42^ Horizontal error bars on the literature values represent the uncertainties as reported in the respective studies, where available. Error bars on the cells from this work represent one standard deviation across the 50 cycles of each cell, and the error bar on the red average represents one standard deviation across the 17 cells. Molecular weights (M.W.) are taken from the experimental sections of the cited studies where reported. N/A indicates that no molar mass was stated.

Despite broad agreement on the synthesis workflow, small differences in precursor handling, base addition rate, workup strategy, and drying can affect product purity and ultimately battery performance. Differences in reported CE, and particularly in how uncertainties are stated, stand out as a critical issue. Fig. 2 summarizes literature data for KCrPDTA negative electrolytes coupled with ferrocyanide/ferricyanide-based positive electrolytes, showing CE ranging from 99.4 to >100%. For comparability, only cells cycled at 100 mA cm^−2^ between 0 and 80% nominal state-of-charge (SOC) in 5 cm^2^ flow cells with carbon paper electrodes are included in this comparison, unless noted otherwise. Upper and lower voltage cut-offs are reported between 1.9–2.1 V and 0.5–1.0 V, respectively. The molar mass assumed in each study is included in Fig. 2. Where stated or inferable, the literature consistently assumed the trihydrate (447.4 g mol^−1^), whereas material stored under ambient conditions is typically the dihydrate (429.4 g mol^−1^). We do not claim that any individual study miscalculated, as the actual hydration state of the isolated material cannot be reconstructed from reported procedures, but assuming the trihydrate for partially dehydrated material would narrow the true SOC window and potentially inflate apparent CE. Reported uncertainties are sometimes calculated across cycles within a single cell rather than across independent experiments, reflecting intra-cell stability rather than inter-cell repeatability.

Following published procedures, our efforts to revalidate KCrPDTA as an RFB negative electrolyte material in a new laboratory involved many cell failures and yielded comparatively lower CE of 99.28 ± 0.14% (10 mL 1 M KCrPDTA negative electrolyte, 30 mL 0.5 M K_4_[Fe(CN)_6_] positive electrolyte, 50 cycles each) across 17 cells with a first-cycle CE of 98.0 ± 0.8% (Fig. 2), consistently lower relative to reported values despite employing the same optimized membranes, buffers, and previously validated cell hardware and testing protocols that include a pre-charge/discharge step to purge residual oxygen and continuous humidified argon sparging of the negative electrolyte. The average voltage efficiency (VE) was 86.6 ± 2.9%, on par with previous reports,^15^ resulting in average energy efficiency (EE) of 85.9 ± 2.8% at 100 mA cm^−2^. Per cycle CE, VE, EE are shown in Fig. S3. Note that cells with average CE over 50 cycles <99% were excluded from this comparison as such values correlated more with cell assembly and components, *e.g.*, batches of membranes, rather than various batches of the active material itself (see section 3.6). The discrepancy in reported and observed CE values highlights the challenges in interlaboratory reproducibility in flow battery research^23^ and underscores the importance of reporting performance metrics across independent cell assemblies rather than single-cell cycling statistics.

### 3.3 Revalidating synthesis

To identify the origin of this discrepancy, the synthesis, work-up, and electrochemical evaluation of KCrPDTA were revisited across multiple precursors, purification strategies, and cell assembly procedures. Briefly, we observed the formation of an amorphous, sticky, crust on top of the hexagonal KCrPDTA crystals upon drying the reaction mixture, after removal of the sulfates, to completion. The stickiness was relatively more pronounced for trials where the initial stage, before adding any base, was performed at lower temperature of 70 °C or for less than 24 h, in agreement with the above discussion regarding coordination polymers.^34^ We therefore adapted the standard protocol for KCrPDTA synthesis from previous reports,^18^ described in the experimental part. Shortly, the ligand is combined with the Cr(iii) source and brought to reflux. The ligand is not initially soluble in this acidic mixture, and the chromium precursor only dissolves slowly, forming a purple solution. Above *ca.* 80 °C the mixture becomes clear and increasingly red in color. After 48 hours, base is slowly added at a defined rate controlled with syringe pumps. This setup allows much more reproducible base addition compared to dripping funnels, where the base solution is also continuously exposed to CO_2_ in air, or addition of solids which cause local pH spikes that may promote olation and the formation of coordination polymers or mixed hydroxo species. The mixture is then cooled to room temperature, and acetone is added to remove K_2_SO_4_ near-quantitatively and the KCrPDTA solution is left to evaporate.

**Fig. 3:**
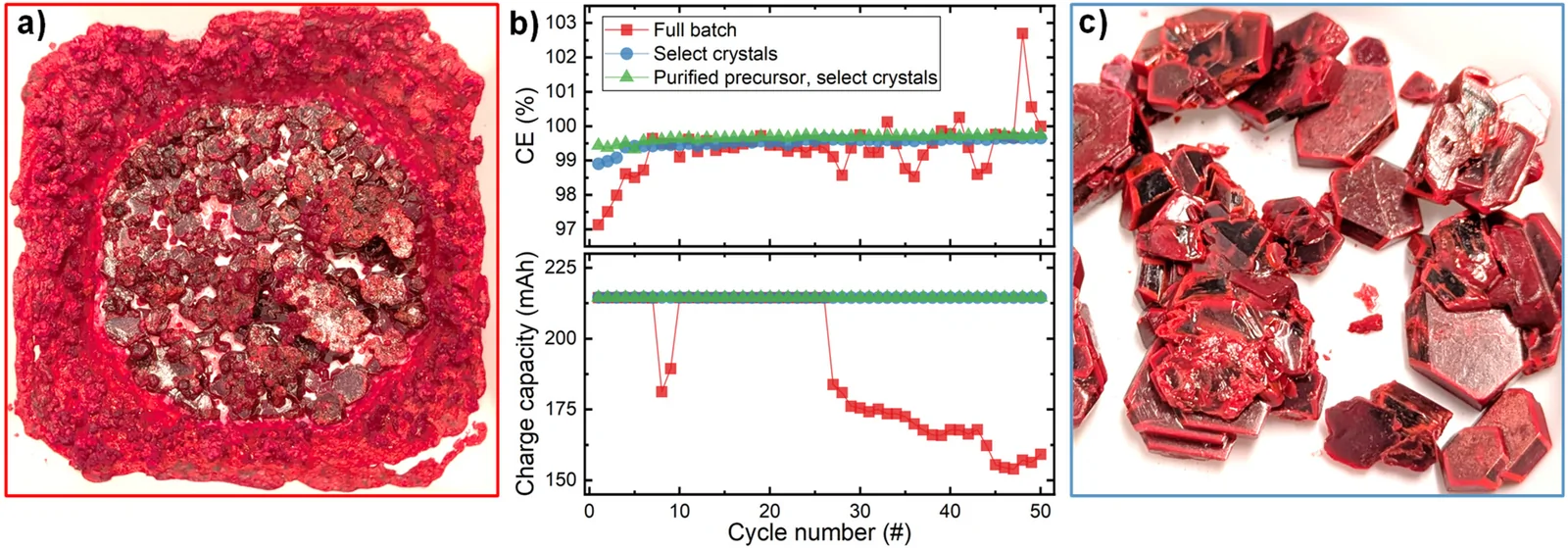
(a) Photograph of a fully evaporated KCrPDTA synthesis batch, showing hexagonal crystals alongside a substantial amorphous crust. (b) Coulombic efficiency (CE) and charge capacity over 50 constant-current cycles for KCrPDTA-K_4_[Fe(CN)_6_] RFBs using negative electrolytes prepared from differently purified material: fully dried batch (red), recrystallized KCrPDTA (blue), and recrystallized KCrPDTA prepared from recrystallized Cr(iii) precursor (green). Cells were cycled between 0 and 80% nominal SOC at ±100 mA cm^−2^. (c) Photograph of selectively harvested KCrPDTA crystals showing the color difference between freshly harvested (dark red, trihydrate) and partially dried crystals (brick red after approximately one day in the fume hood). Full conversion to the dihydrate occurs over roughly one week at room temperature.

We split the product solution into two separate parts. One part was left to fully dry at room temperature, resulting in hexagonal KCrPDTA crystals along with a lighter colored amorphous crust (Fig. 3a). Homogenized *via* grinding and dried for a week at room temperature, the material was then used as RFB negative electrolyte in the same setup as described above. We recorded an average CE over 50 cycles of 99.3% with a first-cycle CE of 97.1%, analogous to our initial attempts, and erratic charge capacity, indicating significant amounts of electrochemically non-inert impurities (Fig. 3b). The second batch was left to evaporate as well, but before drying to completion, individual crystals were removed from the liquor and rinsed with water (Fig. 3c). After grinding and drying at room temperature this sample reached the targeted charge capacity and yielded an average CE over 50 cycles of 99.5% with a first-cycle CE of 98.9%, highlighting the impact of the workup procedure (Fig. 3b). UV–Vis analysis, using the above established molar extinction coefficients, revealed that the full batch sample had an apparent molar mass of 436.0 g mol^−1^, compared to 429.4 g mol^−1^ for KCrPDTA·2H_2_O, indicating an impurity content of approximately 2%, while the recrystallized product was much closer to the expected value (Fig. S4).

As we noticed some discolorations in the Cr(iii) precursor, in multiple batches and irrespective of manufacturer-stated purity, we dissolved the KCr(SO_4_)_2_·12(H_2_O) and filtered off yellow and magnetic grey impurities (Fig. S5). The solution was left to dry at room temperature, and dark purple KCr(SO_4_)_2_·12(H_2_O) crystals were collected and rinsed before the solution was completely evaporated. The evaporating Cr(iii) solution should not be heated above 40 °C, as elevated temperature induces partial substitution of coordinated water by sulfate, forming green sulfato-chromium species. This change in primary coordination sphere alters the speciation of dissolved chromium and can influence subsequent complexation with the ligand, as discussed below. Using the recrystallized Cr(iii) precursor for the synthesis and following the above crystal-picking workup, we observed an average CE over 50 cycles of 99.7% with a first-cycle CE of 99.4% (Fig. 3b), establishing this route as our recommended procedure. Note that these battery experiments were not performed in triplicate to minimize material consumption. Depending on the stringency in crystal selection, the total yield of the synthesis is reduced to 85–90%.

To assess the impact of Cr(iii) speciation, we employed Cr_2_(SO_4_)_3_·6(H_2_O) as chromium-source, which dissolves to form green solutions containing mixtures of inner-sphere sulfato-bound Cr(iii) species rather than the purple hexaaqua complex [Cr(H_2_O)_6_]^3+^.^43^ Following the synthesis procedure described above, the reaction mixture already exhibited a pH of 8.4 after addition of four equivalents of KOH, and only 68.8% of the expected K_2_SO_4_ was recovered during workup. The reaction vessel was coated with a gelatinous layer, and drying yielded only small crystals embedded in a sticky red-brown matrix, and the product yield could not be calculated. UV–Vis spectroscopy indicated an apparent molar mass of *ca.* 515 g mol^−1^, indicating an impurity content of 20% (Fig. S6). During preparation of 1 M negative electrolyte from this material, significant amounts of fine red solids remained indicating large fractions of poorly soluble impurities. The red color indicates that they are not primarily K_2_SO_4_. The material was not cycled in a battery.

The incomplete recovery of K_2_SO_4_ suggests that a portion of sulfate remains incorporated in hydroxo-sulfato-chromium side phases or polymeric species rather than existing as freely dissociated sulfate available for precipitation. The concurrent formation of viscous, amorphous byproducts suggests that Cr(iii) speciation that deviates from hexaqua-complexes promotes side-phase formation that interferes with clean isolation of KCrPDTA.

### 3.4 Revalidating battery performance

Following the above optimized synthesis procedure and selective KCrPDTA crystal isolation, a series of 8 battery experiments was performed employing an electrochemical protocol including various pre-cycling checks, described in detail in the Experimental section. Briefly, the initial area-specific resistance (ASR) was obtained as the inverse of the slope of a linear fit to a linear sweep voltammogram between 1.6 and 1.8 V. This was followed by a charge step to 50% nominal SOC, an electrochemical impedance spectroscopy (EIS) measurement, and full discharge to 0.8 V before commencing the constant-current cycling protocol, consisting of 50 cycles to 80% nominal SOC at ±100 mA cm^−2^ with potential limits of 0.8 and 2.0 V.

**Fig. 4:**
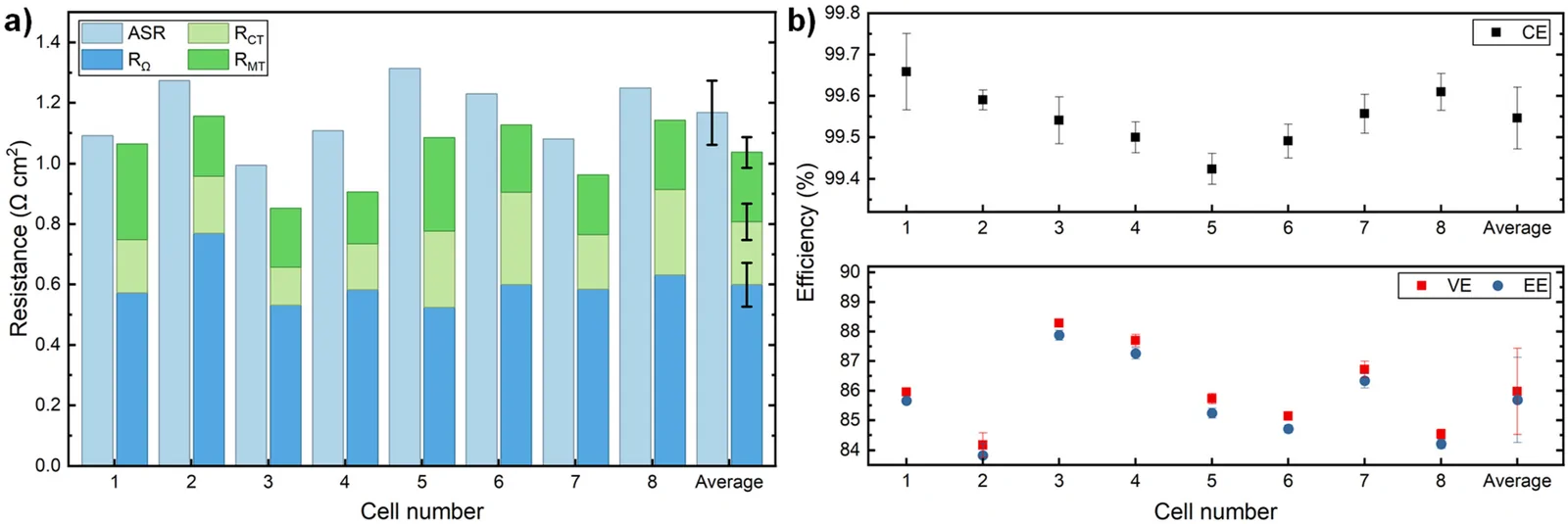
(a) Pre-cycling characterization of eight KCrPDTA-K_4_[Fe(CN)_6_] RFBs. Area-specific resistance (ASR) extracted from a LSV measurement and equivalent circuit resistance contributions, ohmic (*R*_Ω_), charge transfer (*R*_CT_), and mass transport (*R*_MT_), extracted from EIS measurements at 50% nominal SOC prior to constant-current cycling. Error bars represent one standard deviation. (b) Coulombic efficiency (CE) and voltage and energy efficiencies (VE and EE) averaged over 50 constant-current cycles. The cells were cycled between 0 and 80% nominal state-of-charge at ±100 mA cm^−2^. Error bars represent one standard deviation across all cycles per cell.

The EIS data collected at 50% nominal SOC was fitted with an equivalent circuit model commonly employed in flow battery research, comprising an inductor (*L*) in series with an ohmic resistor (*R*_Ω_), followed by two subcircuits connected in series, each consisting of a constant phase element (CPE) in parallel with a resistor, with the high- and low-frequency elements representing charge transfer (*R*_CT_) and mass transport (*R*_MT_) dominated processes, respectively.^18,44,45^ An exemplary Nyquist plot and corresponding equivalent circuit and fit are shown in Fig. S7. The extracted resistance contributions, alongside the directly measured ASR, are summarized across all 8 cells in Fig. 4a. The cells showed comparable resistances, with an average ASR of 1.17 ± 0.11 Ω cm^2^, with *R*_Ω_ being the dominant contributor in all cells, accounting for approximately half the total resistance. The directly measured ASR and the sum of the EIS contributions are distinct measures and are not expected to perfectly coincide. At 50% nominal SOC both redox couples have maximal availability of oxidized and reduced species, so charge-transfer and mass-transport resistances are near their minimum, while the ohmic contribution, which dominates overall resistance, is independent of operating point. The ASR values, on the other hand, are obtained under sustained polarization between 1.6 and 1.8 V where concentration gradients are more developed and reflect the total resistance to driving current under near-operating conditions. We therefore report the directly measured ASR as the operationally relevant resistance and use the EIS decomposition as a diagnostic of its dominant contributions rather than as an independent measure of total resistance.

We obtained an average CE over 50 cycles of 99.55 ± 0.07% with a first-cycle CE of 99.43 ± 0.10% and VE and EE of 86.0 ± 1.5% and 85.6 ± 1.4%, respectively (Fig. 4b). The target capacity was reached in all cycles. The narrow spread in CE reflects the electrochemical reversibility of the active material, while the greater cell-to-cell variability in VE is consistent with inhomogeneities in cell components such as electrodes and membrane, underscoring the need for repeat experiments. ASR broadly anticorrelated with VE, though no definitive trend can be identified across the 8 cells. Taken together, the pre-cycling characterization confirms that cells are sufficiently comparable for meaningful interpretation of the subsequent cycling data. While the efficiencies reported here are lower than select literature values (Fig. 2), the consistency across independent cells provides a statistically grounded benchmark that single-cell studies do not. Given the sensitivity of cell performance to assembly and hardware factors (section 3.6), we consider these values a reliable representation of the intrinsic electrochemical performance of freshly prepared KCrPDTA, against which the performance of reused electrolyte is assessed in the following section.

### 3.5 Reusing KCrPDTA

To assess the intrinsic stability of KCrPDTA, cycled negative electrolytes from well-performing batteries were collected and left to slowly evaporate. Very large KCrPDTA crystals (Fig. S8) were continuously harvested, and the increasingly PDTA-rich liquor was only discarded once its viscosity resembled that of honey. Practically, cycled electrolyte was continuously added to a large beaker and KCrPDTA crystals were gradually harvested, significantly reducing materials consumption for lab-scale experimentation. UV–Vis spectra of fresh, cycled and recrystallized, and twice-recycled material showed minimal changes, supporting the conclusion that KCrPDTA itself is chemically robust under appropriate operating conditions, *i.e.*, below pH ∼13 (Fig. S9).^11^

**Fig. 5:**
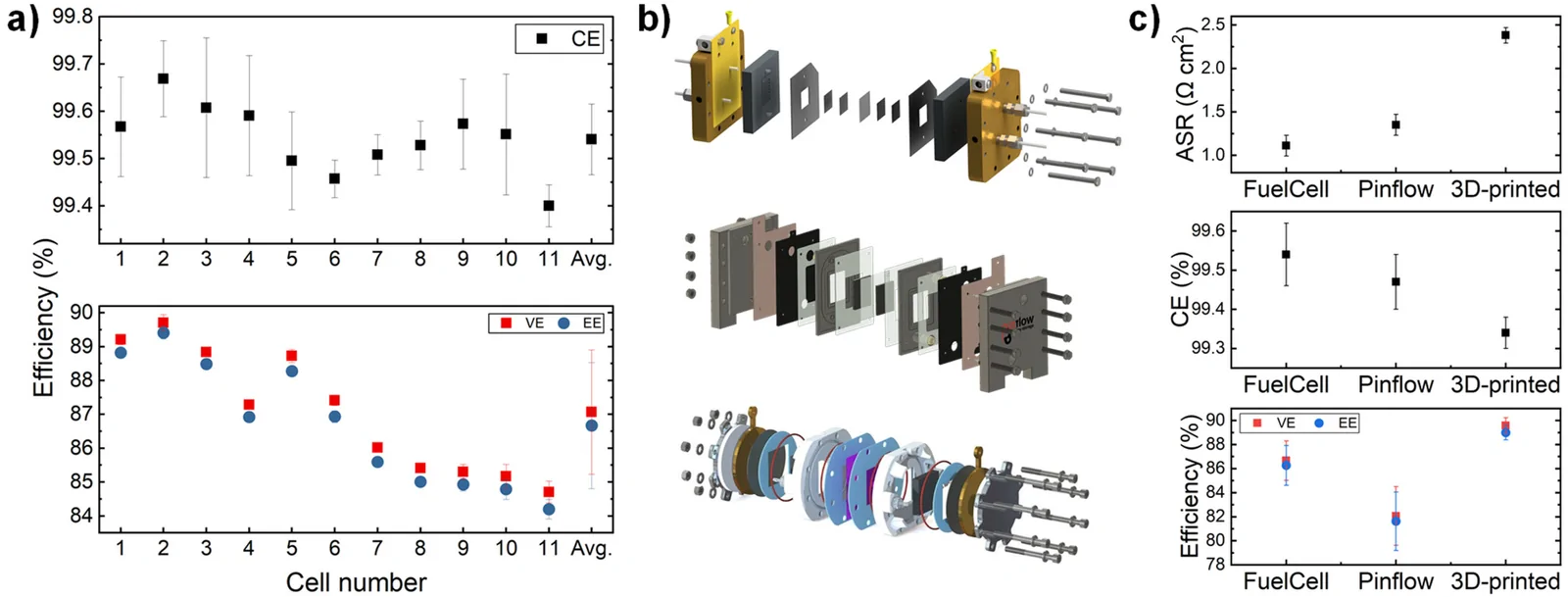
(a) Coulombic efficiency (CE) and voltage and energy efficiencies (VE and EE) of KCrPDTA-K_4_[Fe(CN)_6_] fuel-cell-type RFBs employing reused KCrPDTA, averaged over 50 constant-current cycles. The cells were cycled between 0 and 80% nominal state-of-charge at ±100 mA cm^−2^. Error bars represent one standard deviation across all cycles per cell. (b) Exploded schematic views of the three cell types employed in this work: fuel-cell-type flow cell with serpentine flow field (top), Pinflow cell with interdigitated flow field (middle), and 3D-printed cell with felt electrodes and no flow field (bottom), with the number of cells tested indicated. (c) Comparison of ASR (top), CE (middle), and VE and EE (bottom) across the three cell types. Error bars represent one standard deviation across all cells per architecture. Note that the 3D-printed cells were cycled at 31.25 mA cm^−2^ rather than 100 mA cm^−2^ to maintain comparable cycle duration.

Employing the above-described electrochemical protocol we obtained an average CE over 50 cycles of 99.54 ± 0.07% with a first-cycle CE of 99.25 ± 0.14% across 11 cells with reused KCrPDTA. The average VE was 87.1 ± 1.8% and EE was 86.7 ± 1.9% (Fig. 5a). The average ASR of 1.12 ± 0.11 Ω cm^2^ as well as equivalent circuit resistance contributions agreed with cells using newly synthesized material discussed in section 3.4 (Fig. S10).

To study whether the still-lower CE compared to literature reports may be related to assumed molar masses, which would result in concentration and resulting SOC windows being underestimated by ∼4%, we also performed experiments accessing a narrower SOC window. We employed a new batch of recycled KCrPDTA for these experiments and for fair comparison, we first repeated experiments to 80% nominal SOC, recording average CE of 99.53 ± 0.12%, 86.9 ± 1.2% VE, 86.5 ± 1.3% EE, and first-cycle CE of 99.34 ± 0.12% across 3 cells, on par with previously used material. Cycling to 75% nominal SOC we obtained an average CE over 50 cycles of 99.65 ± 0.04% with a first-cycle CE of 99.48 ± 0.10% across 5 cells (Fig. S11). The average VE and EE were 85.2 ± 1.4% and 84.9 ± 1.4%, respectively. As expected, the 75% nominal SOC cells showed slightly higher CE, a narrower SOC window corresponds to less time at high cell voltage where side reactions such as water reduction can occur, but the improvement of *ca.* 0.1% does still not match some of the reported values in early studies.

The agreement between cells employing freshly synthesized or reused KCrPDTA, spanning 27 independent fuel-cell-type cells across multiple operators and material batches (22 cycled to 80% and five to 75% nominal SOC) demonstrates reproducible and representative performance of KCrPDTA as a flow battery negative electrolyte. The lower efficiencies obtained relative to select literature reports likely reflect the statistical variability inherent to single-cell measurements rather than a fundamental limitation of the material.

### 3.6 Cell design and cell assembly considerations

Beyond material quality, the choice of cell architecture itself has a significant impact on performance (Fig. 5b and c). Following the same protocol as for fuel-cell-type cells, cells from Pinflow energy storage s.r.o. with equivalent electrode area but interdigitated flow fields designed for felt electrodes yielded an average CE over 50 cycles of 99.47 ± 0.08% with a first-cycle CE of 98.7 ± 0.5% and average VE of 80.2 ± 1.1% and EE of 79.8 ± 1.2% across four cells (Fig. S12a). CE was slightly lower compared to the fuel-cell-type cells discussed so far, while VE was reduced by 7%. The average ASR increased by 23.5% to 1.38 ± 0.09 Ω cm^2^ with the contribution of *R*_MT_ being 31% of ASR compared to 20% in fuel-cell-type cells (Fig. S12b), consistent with mass transport limitations imposed by the interdigitated flow field geometry being the primary driver of the reduced VE. Using three instead of two carbon papers on each side of the membrane, as recommended by the manufacturer to reduce contact resistance, reduced the contribution of *R*_MT_ to the ASR of 1.35 ± 0.12 Ω cm^2^ to 27% and yielded average CE of 99.48 ± 0.07%, first-cycle CE of 98.9 ± 0.3%, and average VE of 83.9 ± 1.9% and EE of 83.5 ± 1.8% across four cells (Fig. S12). While VE improved by approximately 4% relative to the two-paper configuration, it remained below fuel-cell-type cell performance, highlighting that lower efficiency metrics may arise from flow field and cell geometry or electrode compression, rather than electrolyte properties or electrochemical protocols.

Using an updated version of the 3D-printed cell design detailed in ref. 23 and 46, which instead of 5 cm^2^ carbon paper employs 16 cm^2^ carbon felts and no flow field, we obtained an average CE over 50 cycles of 99.34 ± 0.04% with a first-cycle CE of 99.0 ± 0.2% across three cells and average VE of 89.6 ± 0.7% and EE of 89.0 ± 0.6% (Fig. S13). The higher ASR of 2.38 ± 0.09 Ω cm^2^ likely reflects the absence of a flow field, which in the other two designs enforces uniform electrolyte distribution and electrode compression, effects that become more pronounced as electrode area increases. The current density was reduced to 31.25 mA cm^−2^ to maintain cycle duration equivalent to the other two cell formats, which likely contributes to improved VE. The number of 3D-printed cells was limited by electrode material availability.

While the cycling protocols and electrode morphologies in all three cell geometries could certainly be optimized towards improved performance, this comparison highlights the impact of cell design on battery performance, even if identical materials and protocols are employed. This is summarized in Table 1 and underscores the importance of appropriate experimental design and reporting for validation studies and benchmarking of cell chemistries. Table 1 also lists our initial pre-optimization results (section 3.2), whose lower and more variable coulombic efficiency quantifies the improvement achieved through the refined workup, hydration control, and cell assembly.

**Table 1:** Per-architecture performance metrics for KCrPDTA-K4[Fe(CN)6] RFBs, all cycled over 50 cycles to 80% nominal state-of-charge at 100 mA cm^−2^ unless indicated. Coulombic, voltage, and energy efficiencies are reported as the mean across all cells of each architecture, with uncertainties denoting one standard deviation. The 3D-printed cells were cycled at 31.25 mA cm^−2^ to maintain comparable cycle duration given their larger electrode area, which contributes to higher voltage efficiency. Cells cycled to 75% nominal SOC are reported separately (Fig. S11)

Cell architecture	Number of cells	Coulombic efficiency (%)	Voltage efficiency (%)	Energy efficiency (%)
Fuel cell prior to optimization	17	99.28 ± 0.14	86.6 ± 2.9	85.9 ± 2.8
Fuel cell	22	99.54 ± 0.08	86.7 ± 1.6	86.3 ± 1.7
Pinflow with two electrodes	4	99.47 ± 0.08	80.2 ± 1.1	79.8 ± 1.2
Pinflow with three electrodes	4	99.48 ± 0.07	83.9 ± 1.9	83.5 ± 1.8
3D printed	3	99.34 ± 0.04	89.6 ± 0.7	89.0 ± 0.6

Beyond cell architecture, assembly quality within a given cell design has a significant impact on performance, and we identified several recurring issues and practical mitigation strategies.

The most impactful finding concerns membrane integrity. Cells exhibiting stable but persistently low CE (90–99%) with no capacity fading or change in charge capacity (Fig. S14) were consistently found to have resistances below 1 kΩ measured prior to filling with electrolyte. We attribute this to electrical shorting across membrane defects such as pinholes, likely exacerbated by uneven membrane compression during assembly. Crucially, this issue was dramatically reduced by sequentially tightening the bolts first to 2 then 4 then 6 Nm, ensuring uniform membrane compression. We therefore highly recommend measuring cell resistances with an ohmmeter and establishing a setup-specific acceptance threshold, in our case >1 kΩ, before committing electrolyte to a cell. Note that applied torque is only an indirect proxy for stack pressure, which also depends on cell geometry and friction, so torque values are best treated as setup-specific.

Flow rate consistency is a second source of cell-to-cell variability that is easy to overlook. Peristaltic pump tubing softens with use, causing flow rates to drift. Since flow rate affects *R*_MT_, uncontrolled variation introduces systematic differences between nominally identical cells. We recommend calibrating flow rates with the cell in-line before every experiment rather than relying on pump settings alone and replacing tubing periodically.^47^ Similarly, compression ratios change as gaskets stiffen over time, altering electrode compression and contact resistance. Regular measurement of gasket thickness before assembly is a simple precaution.

Finally, peristaltic tubing abrasion can introduce debris into the cell, blocking electrode surface area and liquid pathways. We noticed that this differs significantly between pump heads from different manufacturers, and that stretching the tubing manually before clamping the pump head shut significantly reduces particulate release.

Taken together, these considerations, routine resistance checks, in-line flow calibration, controlled mechanical assembly, and tubing maintenance, are straightforward to implement and can prevent the misattribution of hardware artefacts to electrolyte properties, which is particularly important when evaluating new RFB electrolytes where performance benchmarks are still being established.

## 4. Conclusion

Reproducing performance metrics of KCrPDTA negative electrolytes proved challenging, requiring systematic identification of variability sources. Recrystallization of the Cr(iii) precursor and selective KCrPDTA crystal isolation during workup are critical improvements, and the hydration state of the isolated material must further be accounted for when preparing electrolytes gravimetrically, as molar mass differences between the di- and trihydrate of KCrPDTA directly propagate into SOC and coulombic efficiency misinterpretations.

Employing a standardized protocol, we obtained a highly reproducible average coulombic efficiency of 99.54 ± 0.08% and energy efficiency of 86.3 ± 1.7% over 50 cycles to 80% nominal SOC at 100 mA cm^−2^ across 22 fuel-cell-type KCrPDTA/K_4_[Fe(CN)_6_] cells assembled by multiple operators using various material batches. Across all four architectures in this study (38 cells), coulombic efficiency ranged from 99.3 to 99.7%, while energy efficiency varied from roughly 80 to 86% at 100 mA cm^−2^, with the 3D-printed cells reaching 89% at a lower current density. This represents the most statistically robust reproducibility assessment reported for this system to date. The consistent performance of KCrPDTA recovered by recrystallization from used negative electrolytes confirms the intrinsic chemical robustness of the chelate and provides a practical route to reducing material consumption in laboratory studies.

Cell architecture and assembly further contribute to performance variability. A defined bolt-tightening sequence and setup-specific cell resistance threshold before filling with electrolyte, as well as routine flow rate calibration are straightforward precautions that prevent hardware artefacts from being misattributed to electrolyte properties. Coupled with freely available raw data, transparent analysis, and comprehensive metadata, these results establish a foundation for meaningful benchmarking of RFB electrolytes. Structured, machine-readable datasets of this kind enable direct cross-study comparison and allow re-analysis as methods and reporting standards evolve, providing a framework for assessing RFB performance more broadly. Together with the reporting template introduced here, this represents a step toward the structured reporting standards already established for other battery types.

## Author contributions

G. K.: formal analysis, writing – original draft. Q. L.: investigation. J. A.: investigation. L. C.: investigation. R. C.: investigation. T. E.: investigation. B. V.: investigation. C. B.: writing – review & editing, funding acquisition. D. R.: conceptualization, methodology, investigation, formal analysis, writing – original draft, writing – review & editing, supervision, funding acquisition.

## Conflicts of interest

There are no conflicts of interest to declare.

## Supplementary Material

EB-OLF-D6EB00156D-s001

## Data Availability

The data supporting the findings of this study are openly available in a Zenodo repository at https://doi.org/10.5281/zenodo.20338408. A large dataset comprising cycling data for 62 RFB cells, including early experiments with suboptimal performance due to material impurities or cells with assembly artefacts is provided in a form compliant with the Battery Data Format. Semantically annotated metadata compliant with the BattINFO ontology is provided. Code availability: The ‘PyFlowBatt’ analysis source code is available at https://github.com/empaeconversion/PyFlowBatt and is installable from the Python package index (PyPI) with ‘pip install PyFlowBatt’. The ‘BattINFO converter’ app source code is available at https://github.com/EmpaEconversion/BattInfoConverter, it is installable from PyPI with ‘pip install battinfoconverter-backend’, and the app can be accessed on streamlit at https://battinfoconverter.streamlit.app/, where the redox flow battery template is available on the downloads page. Supplementary information (SI): mass spectrometry of TGA-products, UV–Vis calibration, per-cycle data of initial cell tests, UV–Vis analysis and optical characterization of impurities and precursors, photographs of KCrPDTA crystals, impedance fitting results, per-cycle data of various cell types, and cycling data of shorted cells with cell resistance <1 kΩ. See DOI: https://doi.org/10.1039/d6eb00156d.
