# Supplementary material for: Revalidating KCrPDTA for aqueous redox flow batteries
Source: EES Batter. 2026 Sep 14. Online ahead of print. doi: 10.1039/d6eb00156d (PMC13583517; doi:10.1039/d6eb00156d)
Supplement: Supplementary file 1 [file EB-OLF-D6EB00156D-s001.pdf]

Supplementary Information (SI) for EES Batteries.
This journal is © The Royal Society of Chemistry 2026

Supporting Information for

Revalidating KCrPDTA for aqueous redox flow batteries

Graham Kimbell,[1#] Qirui Liu,[1,2#] Jeonghoon Ahn,[1,3] Lina Camenisch,[1] Rohat Celik,[1] Talia Echeverria,[1] Baban Deep Virk,[1] Corsin Battaglia,[1,2,4,5] David Reber[1*]

1) Empa, Swiss Federal Laboratories for Materials Science and Technology, 8600 Dübendorf, Switzerland
2) Department of Materials, ETH Zürich, Zürich, 8093, Switzerland
3) Department of Chemistry, Biochemistry and Pharmaceutical Sciences, University of Bern, Bern, 3012, Switzerland
4) Department of Information Technology and Electrical Engineering, ETH Zurich, Zurich, 8092, Switzerland
5) School of Engineering, Institute of Materials, EPFL, Lausanne, 1015, Switzerland

# Equal contribution

*E-mail: david.reber@empa.ch

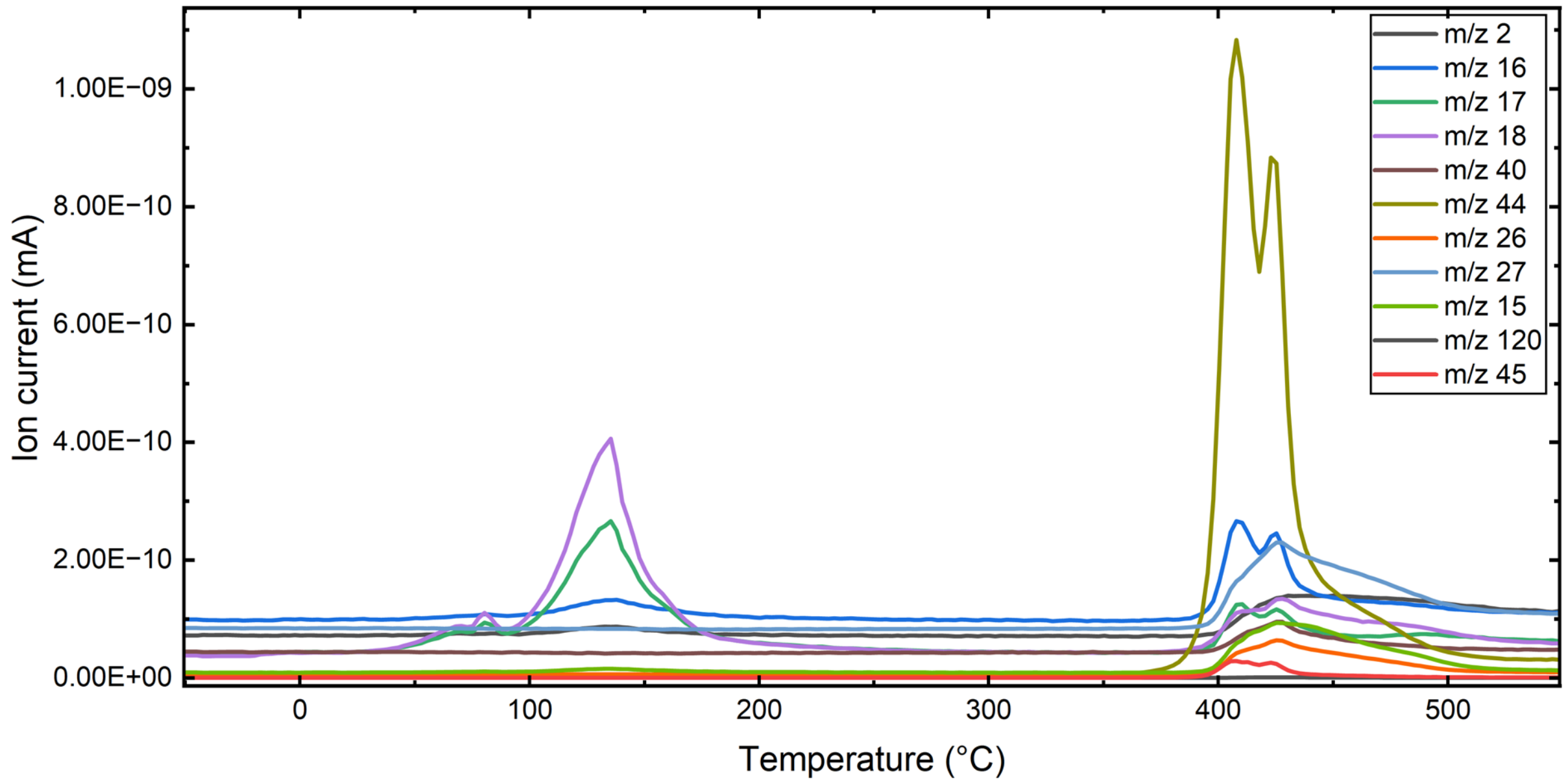

Figure S1. Mass spectrometry signal for H_2O (m/z = 18) and CO_2 (m/z = 44) recorded simultaneously during TGA of KCrPDTA trihydrate, confirming that the mass loss below 370 °C corresponds to lattice water release rather than ligand decomposition.

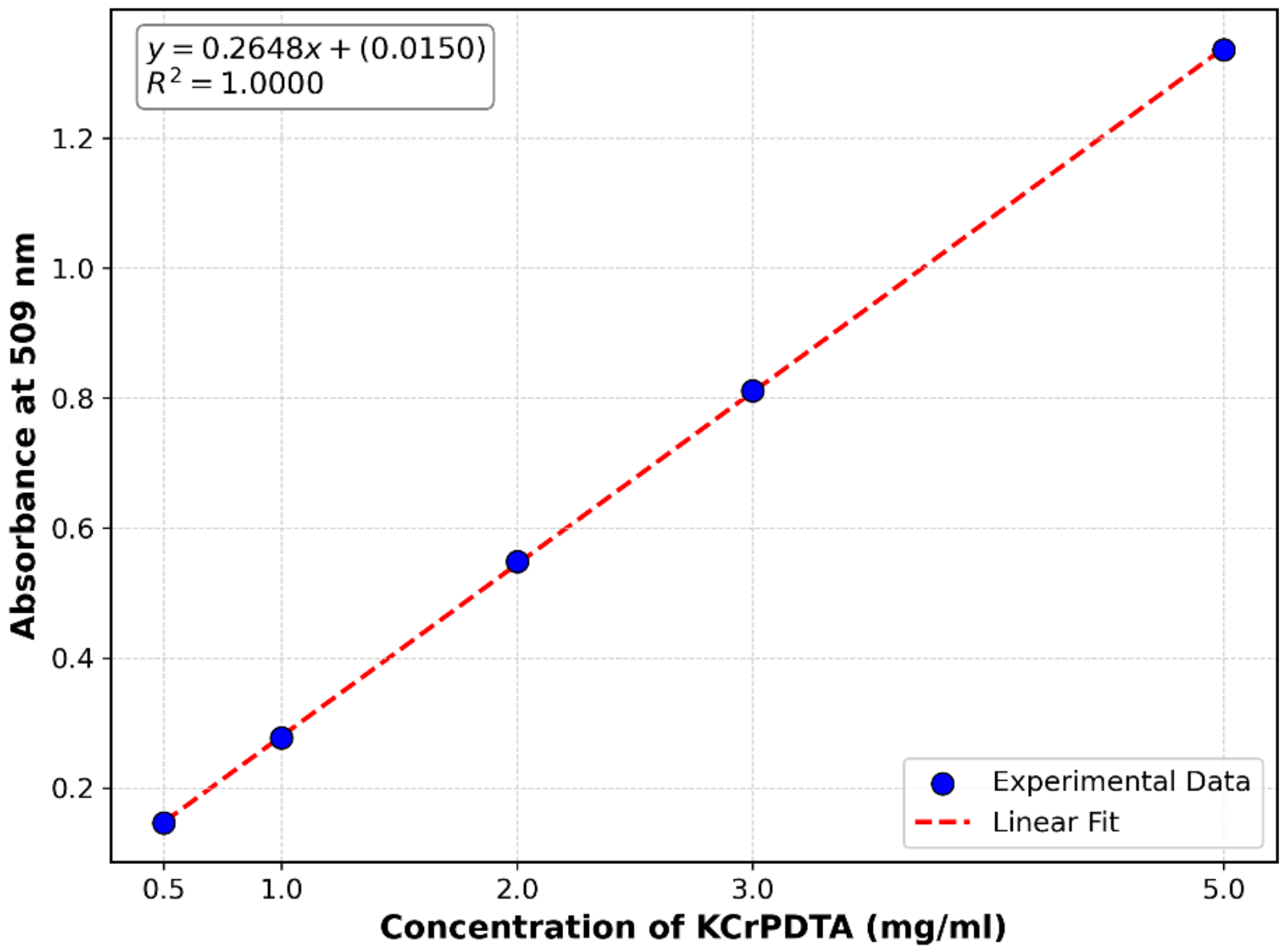

Figure S2. Calibration series confirming Beer-Lambert linearity for KCrPDTA at 509 nm (dihydrate, ultrapure water, pH 6, 1 cm path length). R^2 = 1.000. The molar absorptivity reported in the main text was determined from 2 mg mL^{-1} solutions.

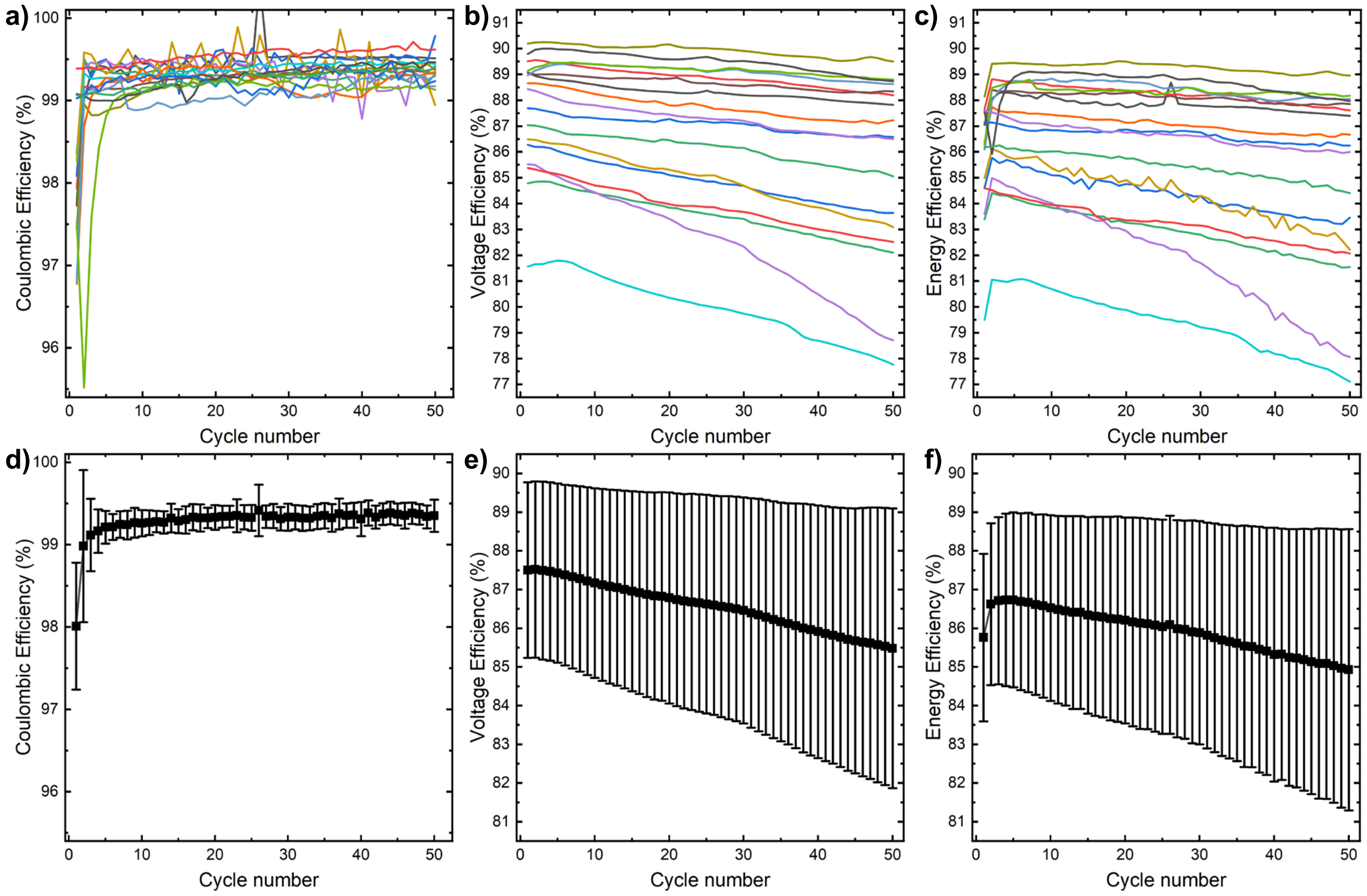

Figure S3. a, d) Per-cycle average Coulombic efficiency (CE), **b, e)** voltage efficiency (VE), and **c, f)** energy efficiency (EE) for the 17 KCrPDTA-$K_4Fe(CN)_6$ RFBs shown in Figure 2b, assembled following published procedures prior to our own efforts. Cells were cycled between 0 and 80% theoretical SOC at ±100 mA cm^{-2}. Error bars represent one standard deviation.

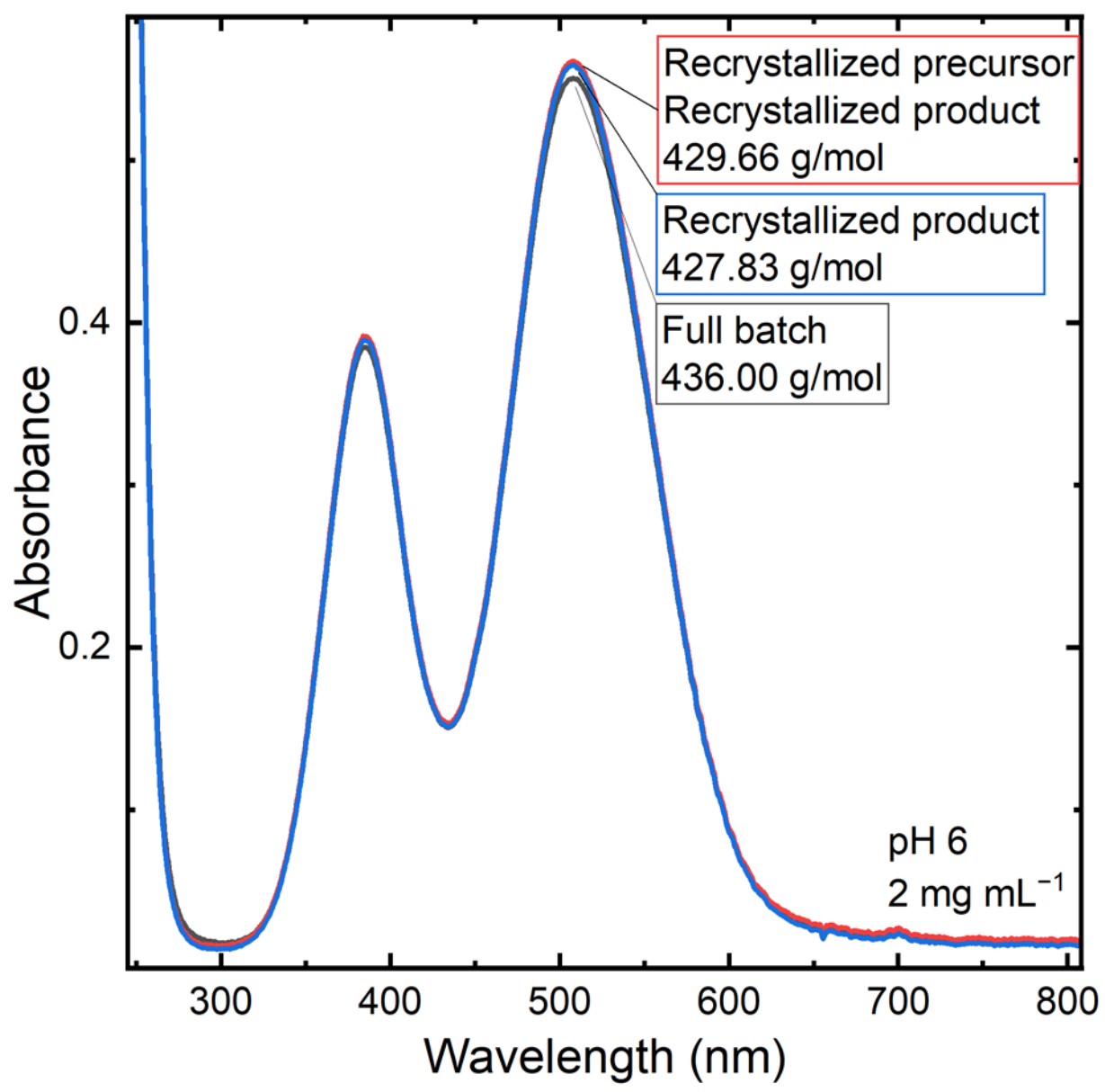

Figure S4. UV-Vis absorption spectra of 2 mg mL^{-1} aqueous solutions prepared from the fully dried synthesis batch and the selectively recrystallized KCrPDTA batches. The apparent molar mass derived from the absorbance at 509 nm indicates an impurity content of approximately 2% in the fully dried batch.

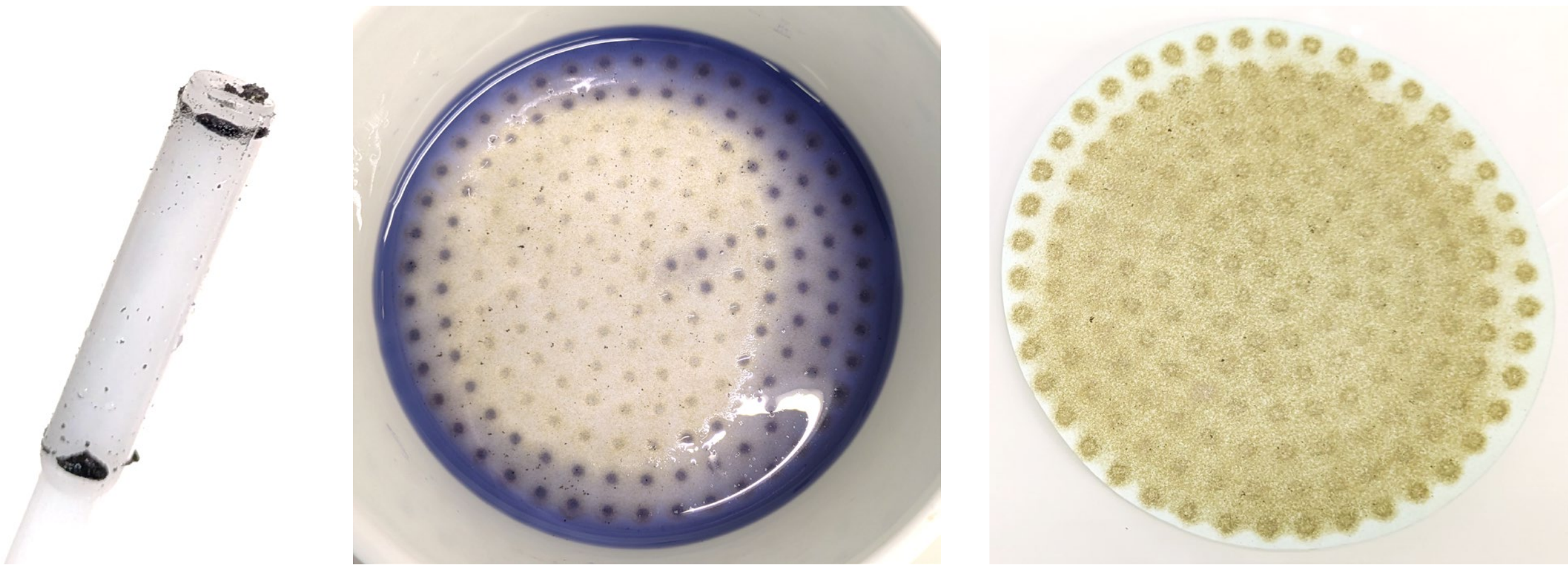

Figure S5. Photographs of impurities isolated by filtration of $KCr(SO_4)_2 \cdot 12H_2O$ solutions, showing magnetically active grey and yellow impurities.

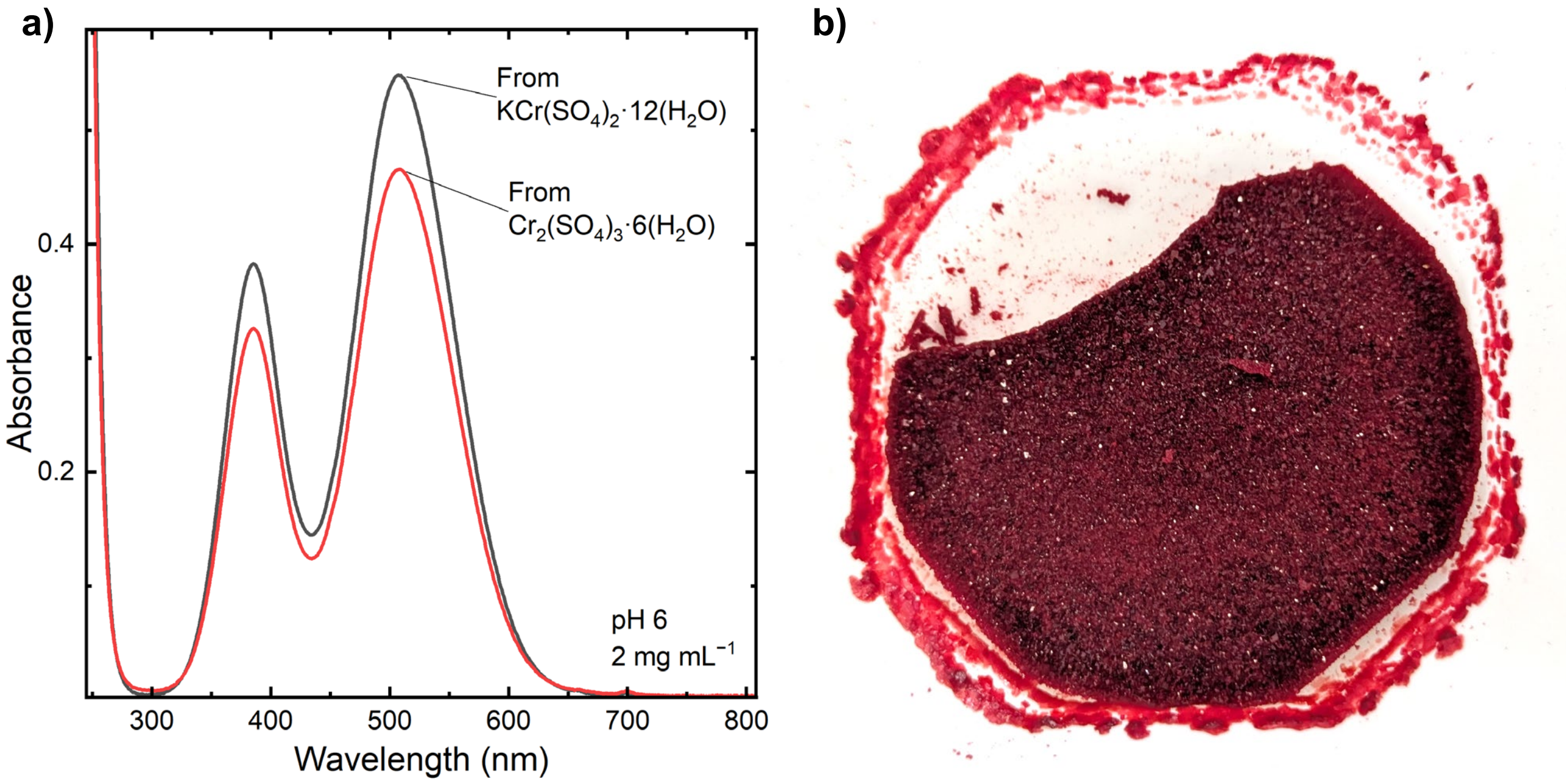

Figure S6. a) UV-Vis absorption spectrum of 2 mg mL^{-1} aqueous solution prepared from the KCrPDTA synthesis product obtained using $Cr_2(SO_4)_3 \cdot 6(H_2O)$ as precursor. The apparent molar mass of ~515 g mol^{-1} indicates an impurity content of approximately 20% relative to pure KCrPDTA $\cdot$ $2H_2O$. b) Photograph of the as obtained product, exhibiting very small crystallites surrounded by a sticky matrix.

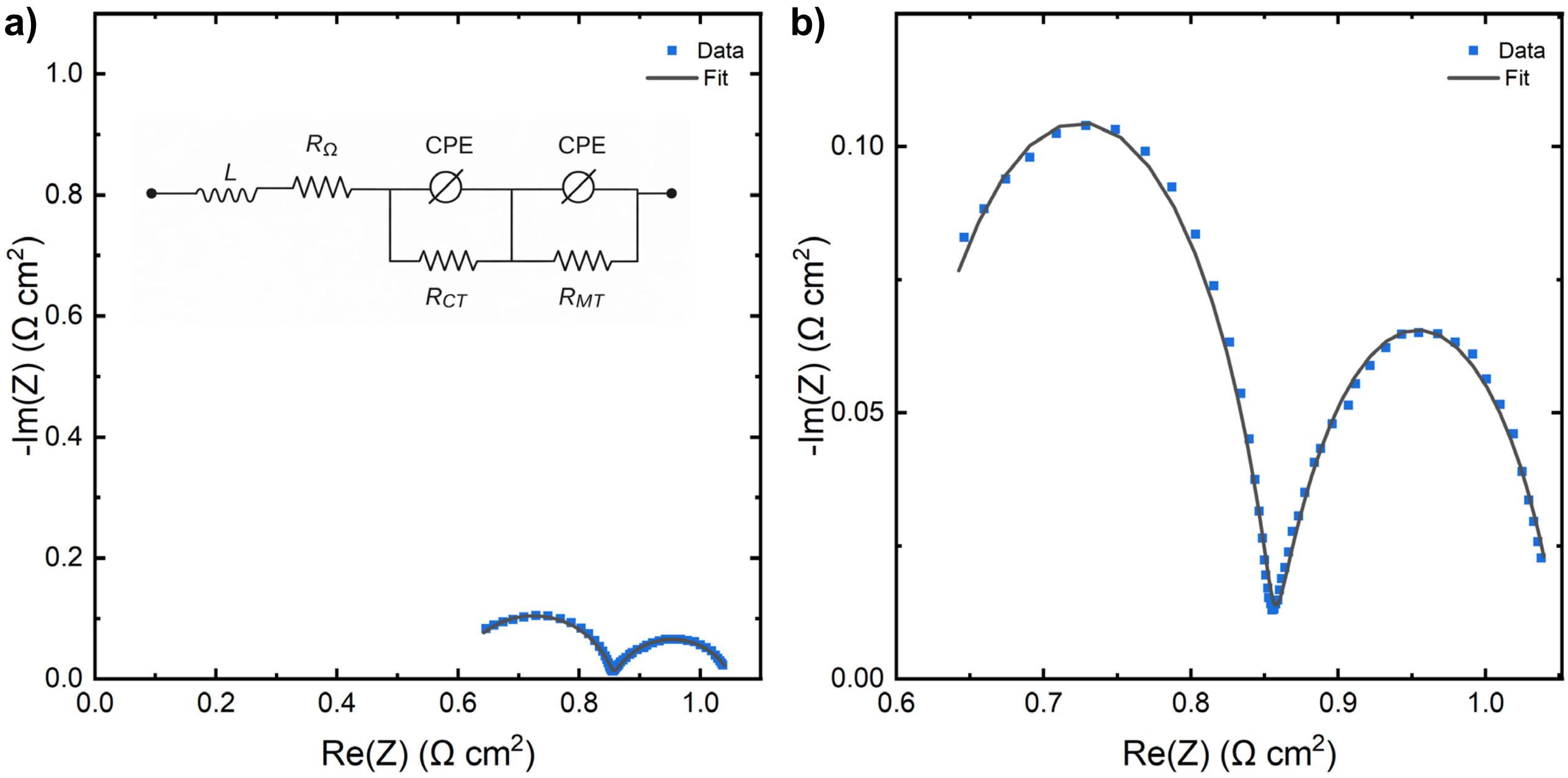

Figure S7. a) Representative Nyquist plot and equivalent circuit fit for a KCrPDTA-$K_4Fe(CN)_6$ RFB using freshly synthesized material, recorded at 50% SOC prior to galvanostatic cycling. The equivalent circuit model comprises an inductor (L) in series with an ohmic resistor (R_{Ω}), followed by two subcircuits each consisting of a constant phase element (CPE) in parallel with a resistance representing charge transfer (R_{CT}) and mass transport (R_{MT}) processes. **b)** Expanded view of the same spectrum.

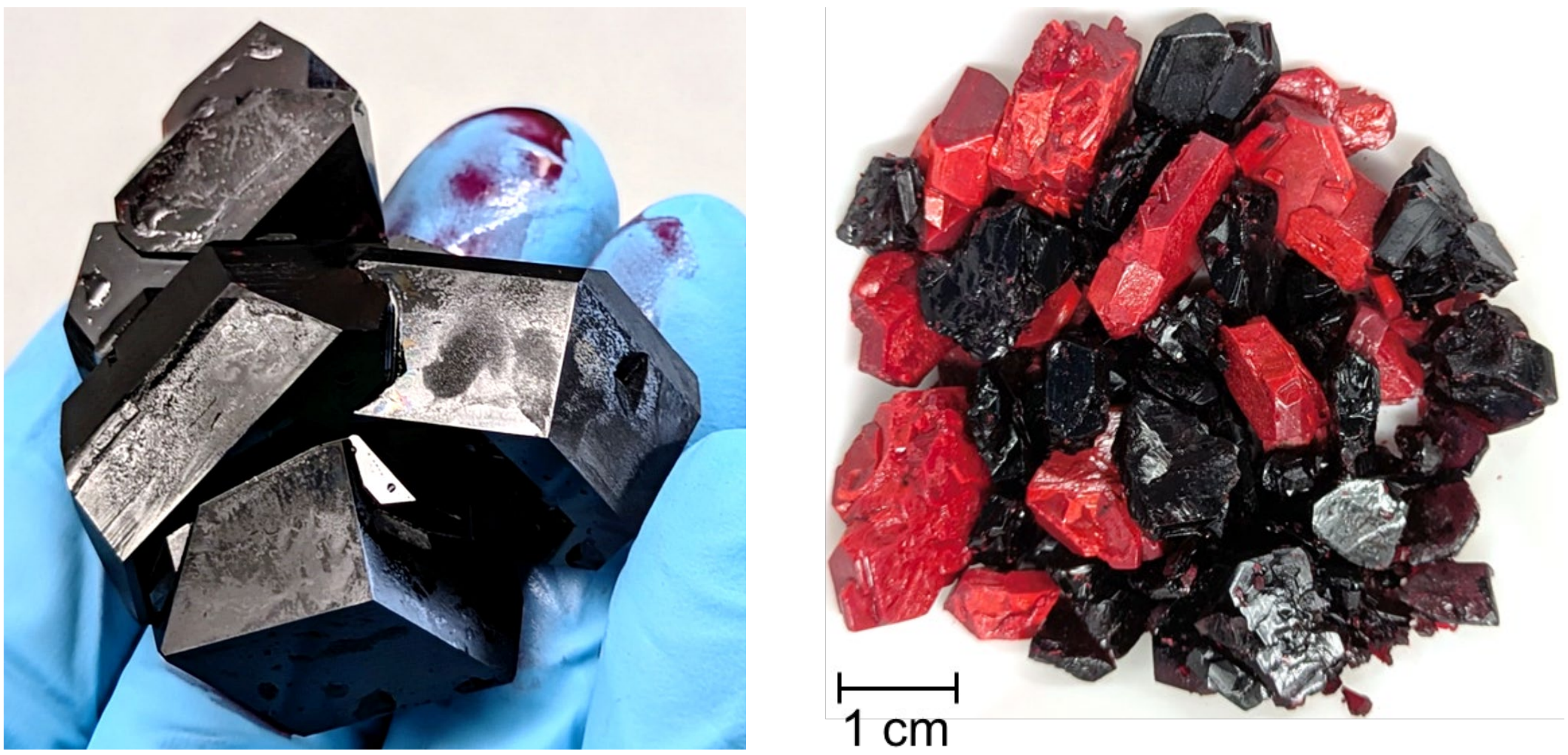

Figure S8. Photographs of large KCrPDTA crystals harvested by evaporative recrystallization from cycled negative electrolyte. Crystal dimensions of several centimeters are routinely obtained, facilitating selective isolation.

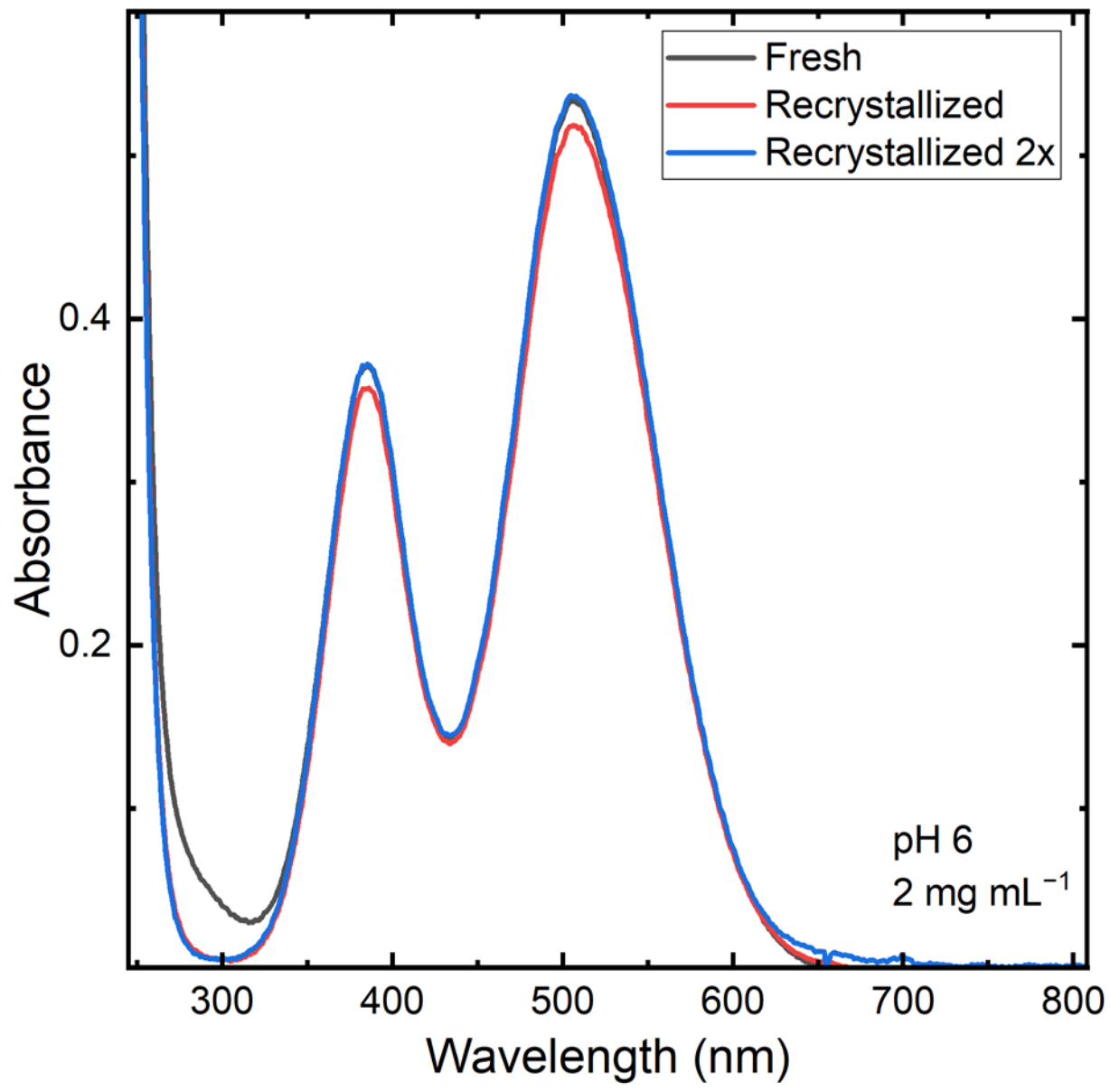

Figure S9. UV-Vis absorption spectra of 2 mg mL^{-1} aqueous solutions of fresh KCrPDTA, KCrPDTA recovered and recrystallized after one cycling campaign, and KCrPDTA recovered after a second cycling campaign. The minimal spectral differences support the chemical robustness of the $CrPDTA^{-}$ chelate under cycling conditions below pH ~13.

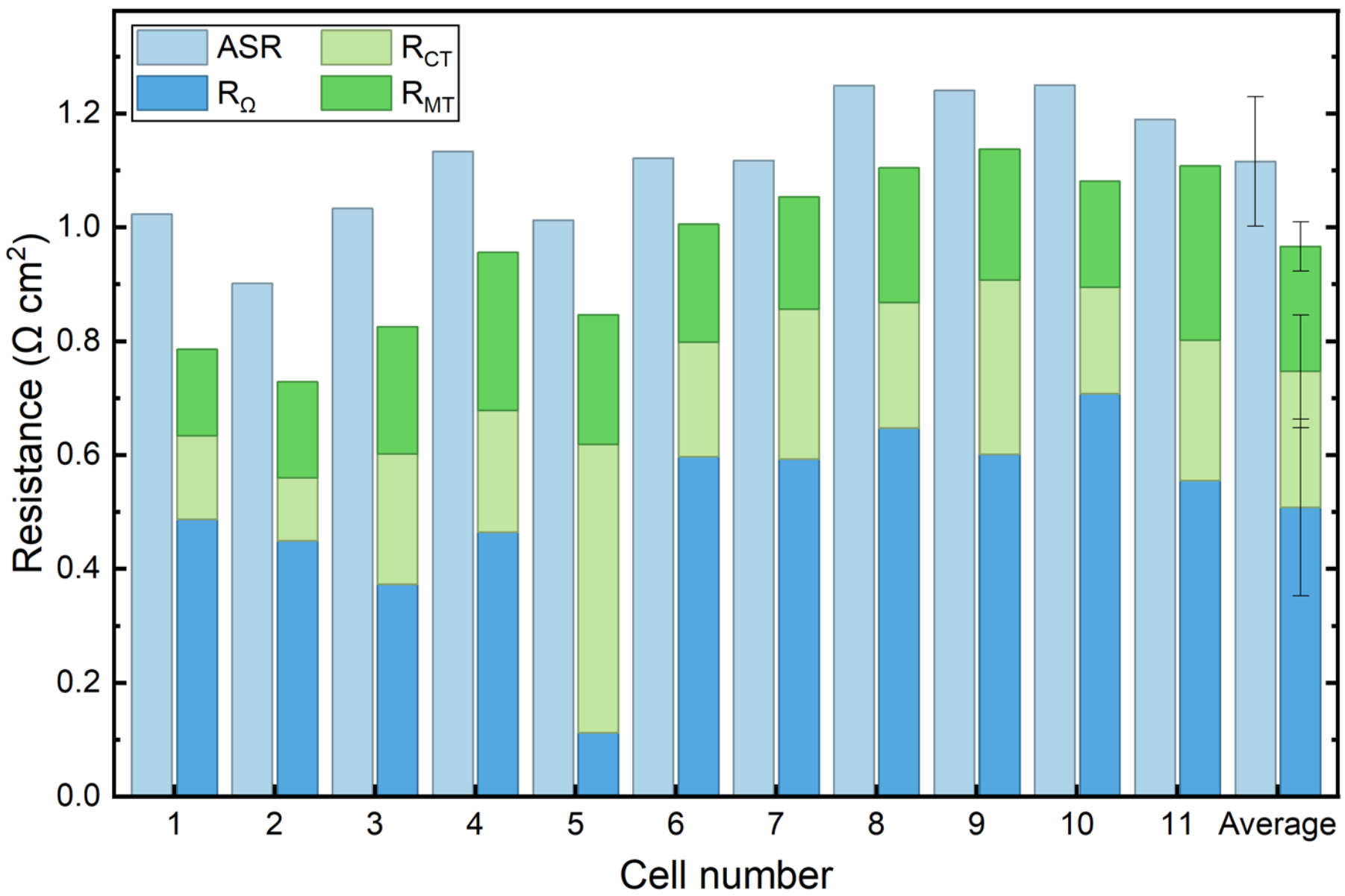

Figure S10. Pre-cycling characterization of eleven KCrPDTA-$K_4Fe(CN)_6$ RFBs using recycled KCrPDTA. Area-specific resistance (ASR) extracted from a LSV measurement and equivalent circuit resistance contributions, ohmic (R_Ω), charge transfer (R_{CT}), and mass transport (R_{MT}), extracted from EIS measurements at 50% SOC. Error bars on the average represent one standard deviation.

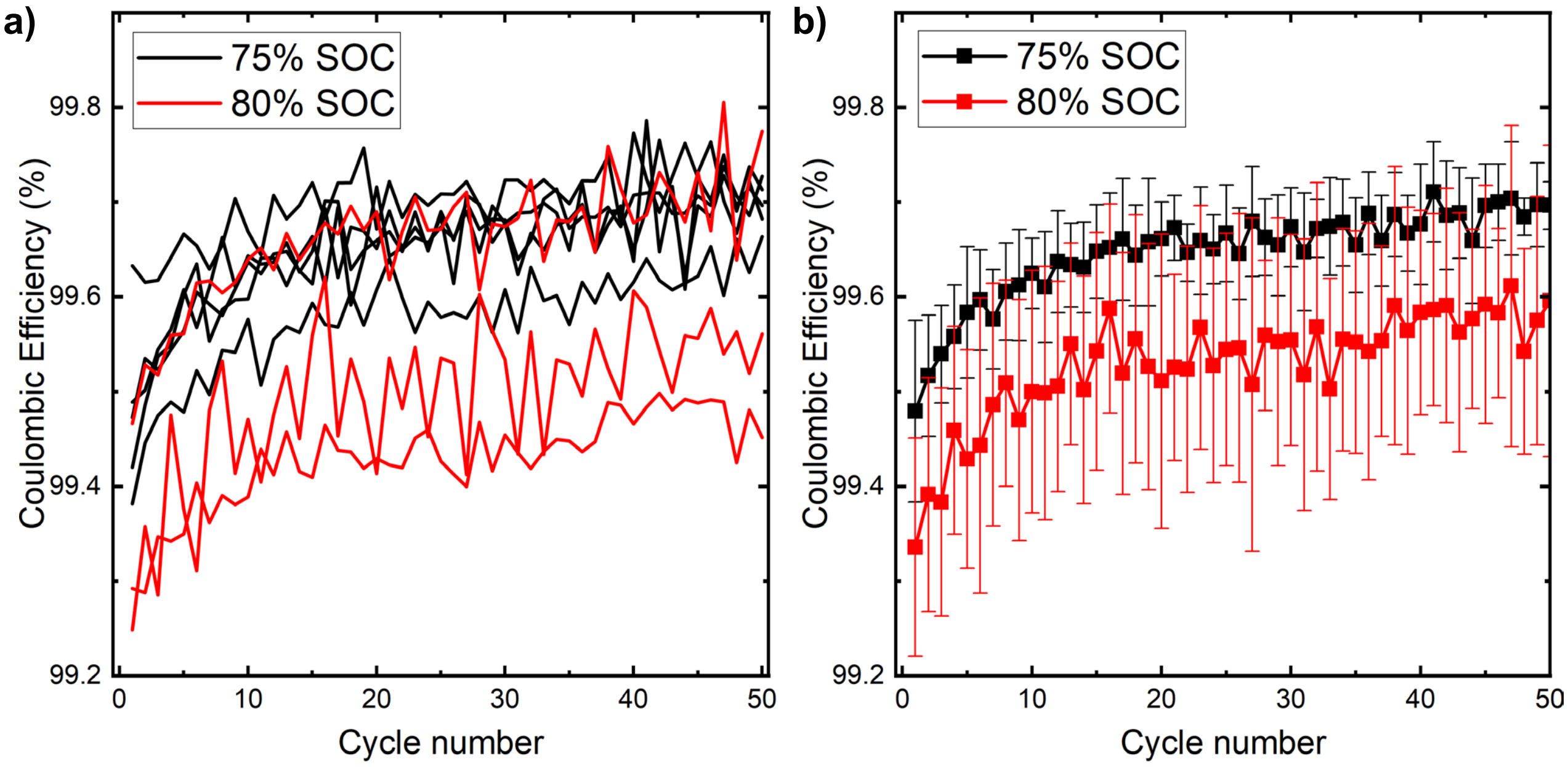

Figure S11. Coulombic efficiency (CE) of KCrPDTA-$K_4Fe(CN)_6$ RFBs cycled to 75% and 80% theoretical SOC using re-used KCrPDTA. **a)** CE of individual cells. **b)** Mean CE with one standard deviation for each SOC. Cells were cycled at ±100 mA cm^{-2} with cell voltage limits of 0.8 and 2.0 V.

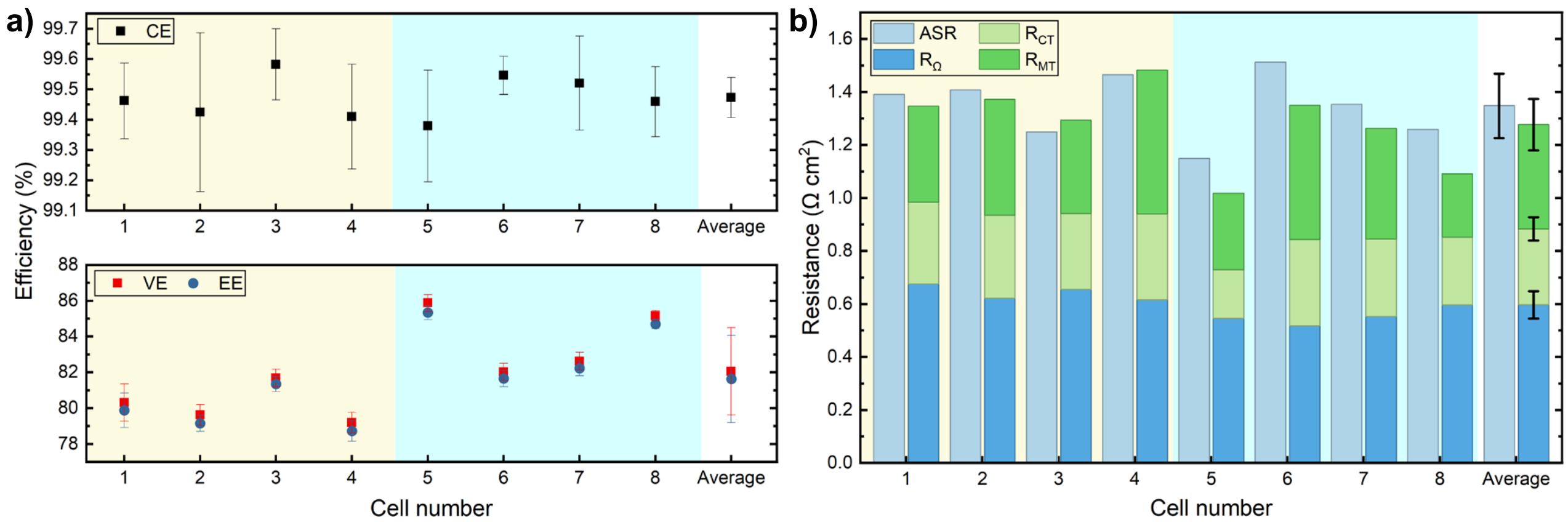

Figure S12. Electrochemical performance of KCrPDTA-$K_4Fe(CN)_6$ RFBs in Pinflow cells with interdigitated flow field. Yellow background: two carbon paper electrodes per side; blue background: three carbon paper electrodes per side, as recommended by the manufacturer to reduce contact resistance. **(a)** CE (top) and VE and EE (bottom) averaged over 50 galvanostatic cycles at ±100 mA cm^{-2}. Error bars represent one standard deviation across all cycles per cell. **(b)** ASR and equivalent circuit resistance contributions (R_Ω, R_{CT}, R_{MT}) extracted from EIS measurements at 50% SOC. Error bars on the average represent one standard deviation.

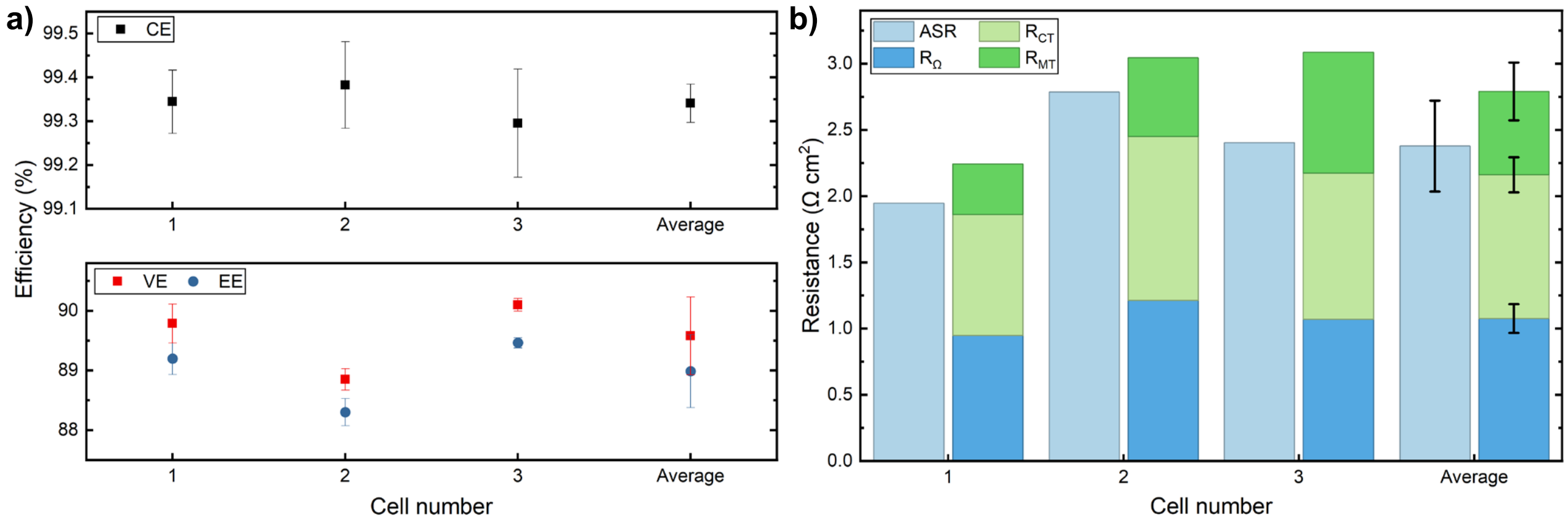

Figure S13. Electrochemical performance of KCrPDTA-$K_4Fe(CN)_6$ RFBs in 3D-printed flow cells with 16 cm^2 carbon felt electrodes and no flow field. **(a)** CE (top) and VE and EE (bottom) averaged over 50 galvanostatic cycles at ±31.25 mA cm^{-2}. Error bars represent one standard deviation across all cycles per cell. **(b)** ASR and equivalent circuit resistance contributions (R_{Ω}, R_{CT}, R_{MT}) extracted from EIS measurements at 50% SOC. Error bars on the average represent one standard deviation.

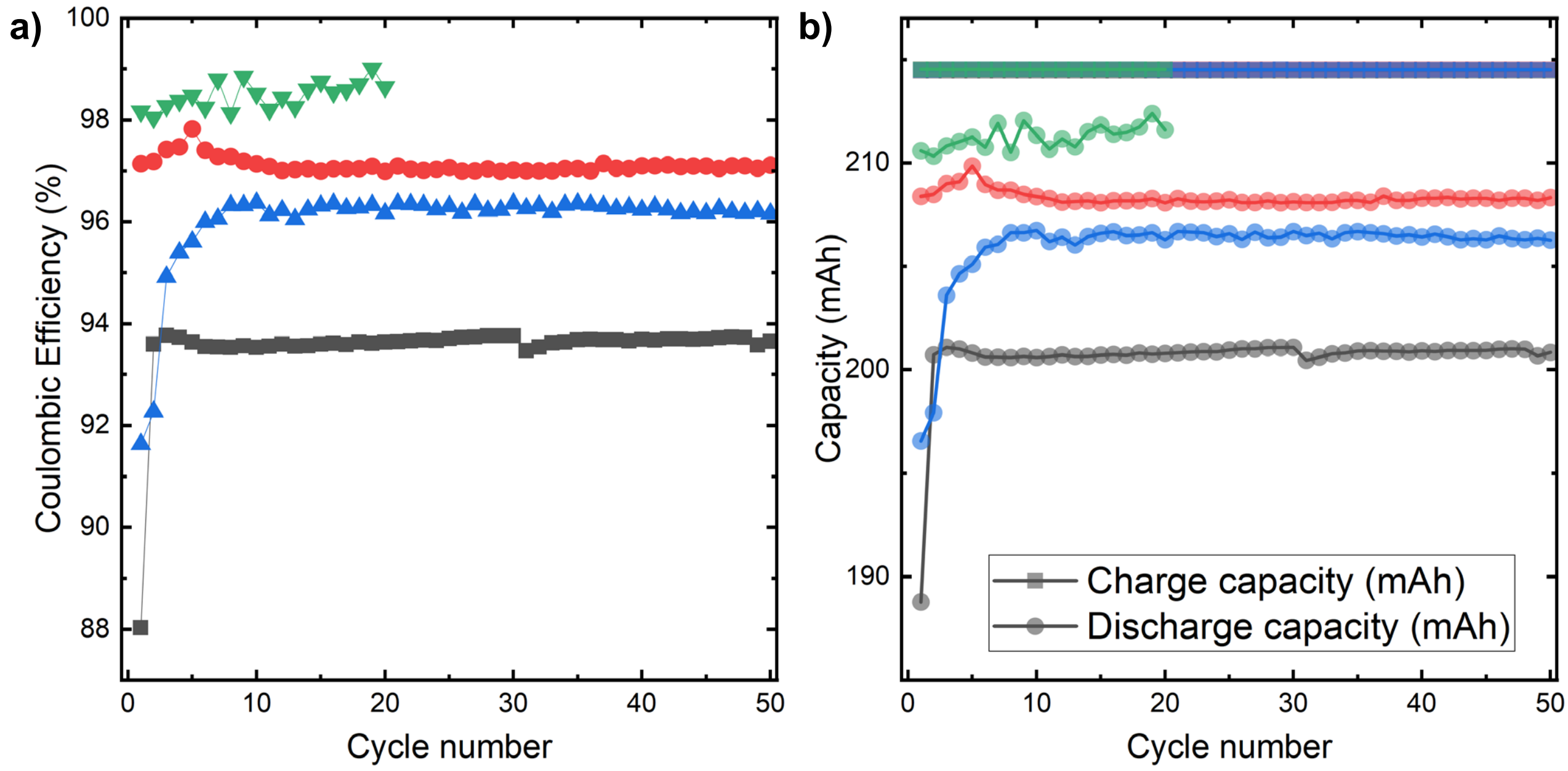

Figure S14. Representative cycling data for KCrPDTA-$K_4Fe(CN)_6$ RFBs exhibiting persistently low but stable CE. **a)** CE and **b)** charge and discharge capacity over 50 cycles. The different colors/symbols represent individual cells. The absence of capacity fading and the stability of charge capacity indicate that the CE loss does not arise from faradaic side reactions such as hydrogen evolution but is instead consistent with electrical shorting across membrane defects. All cells shown had inter-collector resistances below 1 kΩ prior to filling with electrolyte.
